# Grid-Robust Efficient Neural Interface Model for Universal Molecule Surface Construction from Point Clouds

**DOI:** 10.1021/acs.jpclett.3c02176

**Published:** 2023-10-02

**Authors:** Yongxian Wu, Haixin Wei, Qiang Zhu, Ray Luo

**Affiliations:** † Departments of Chemical and Biomolecular Engineering, Molecular Biology and Biochemistry, Materials Science and Engineering, and Biomedical Engineering, University of California, Irvine, California 92697, United States; ‡ Department of Chemistry and Biochemistry, University of California, San Diego, California 92093, United States

## Abstract

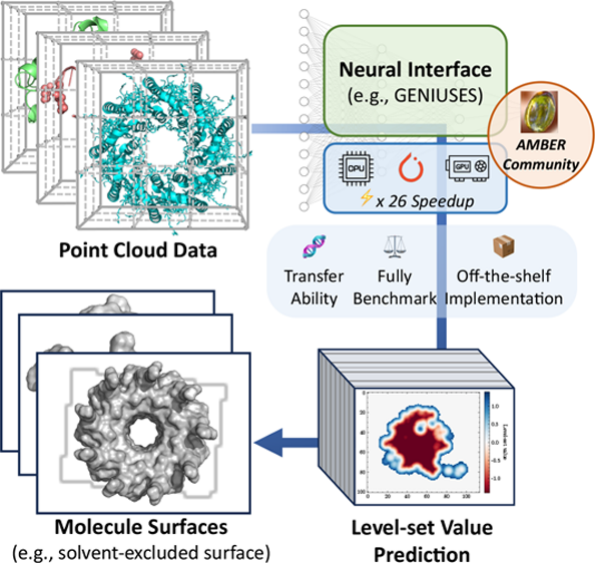

Molecular surfaces play a pivotal role in elucidating the properties and functions of biological complexes. While various surfaces have been proposed for specific scenarios, their widespread adoption faces challenges due to limited efficiency stemming from hand-crafted modeling designs. In this work, we proposed a general framework that incorporates both the point cloud concept and neural networks. The use of matrix multiplication in this framework enables efficient implementation across diverse platforms and libraries. We applied this framework to develop the GENIUSES (**G**rid-robust **E**fficient **N**eural **I**nterface for **U**niversal **S**olvent-**E**xcluded **S**urface) model for constructing SES. GENIUSES demonstrates high accuracy and efficiency across data sets with varying conformations and complexities. Compared to the classical implementation of SES in the AMBER software package, our framework achieved a 26-fold speedup while retaining ∼95% accuracy when ported to the GPU platform using CUDA. Greater speedups can be obtained in large-scale systems. Importantly, our model exhibits robustness against variations in the grid spacing. We have integrated this infrastructure into AMBER to enhance accessibility for research in drug screening and related fields, where efficiency is of paramount importance.

Accurate and efficient representation of surfaces holds critical significance in various fields, including enzymology, rational drug design, and molecular recognition,^1^ as well as in interpreting physical properties like the partition coefficient, solubility, and rate constants.^2,3^ Over the past few decades, numerous methods have been introduced for specific applications, such as the solvent-accessible surface (SAS),^4,5^ solvent-excluded surface (SES),^6,7^ and van der Waals (vdW) surface.^6,8,9^ For instance, the SAS method, derived initially from the solvent-accessible area, is used to study the protein folding problem,^4,6^ while the SES boundary in the Poisson–Boltzmann-based solvent model led to physically meaningful results in the computation of reaction field energies and potential of mean forces.^10−12^

In pursuit of accuracy and efficiency, extensive efforts have been directed toward the development of analytical solutions for surface generation.^13,14^ Distinct strategies and programs have been tailored to specific surface generation scenarios.^15−18^ For example, a refined density function strategy founded on a modified vdW surface was suggested for numerical Poisson–Boltzmann applications.^19^ Taking both accuracy and efficiency into consideration, an analytic surface representation was generated in advance and then mapped onto arbitrary lattices.^13,14,20^ Such strategy and algorithm were further optimized and streamlined by Rocchia et al.^21^ Concurrently, the field-view method was utilized for SES or SAS generation under the finite-difference scheme.^22^ Yet, significant challenges persist due to the time-consuming process and complexities in determining adequate surface curvatures and higher-order surface parameters for implicit solvent simulations. Additionally, results are sensitive to grid discretization.^23,24^ It is worth noting that a surface-free Poisson–Boltzmann solver model treats the solute and solvent uniformly, bypassing the necessity of generating a molecular surface.^25^ The level set function, a mathematical tool leveraged in computer graphics, has displayed versatility in shape representation and analysis.^26,27^ Although the efficiency has improved compared with analytical algorithms, it is still far from ready for deployment in drug screening in terms of speed. Lately, the application of machine learning techniques has garnered increasing attention due to their flexibility and efficiency in fitting given a sufficient number of data samples. Successes have been documented in various disciplines, including chemistry and physics.^27−35^ Considering the rapidly improving computational performance of hardware (e.g., TPU (tensor processing unit) and GPU (graphics processing unit)), the enhanced utilization of these techniques is projected to boost efficiency.

Taking the SES as a representative example, due to its complexity, we integrated the level set function with machine learning techniques to balance accuracy and efficiency in surface generation. Contrary to our previous surface generation method, MLSES,^27^ we proposed a novel framework that can be generalized for any surface generation without requiring specific expert knowledge (Figure 1). The core of this method lies in matrix manipulation, which enables easy transfer across various platforms and libraries. A model named GENIUSES (grid-robust efficient neural interface for universal solvent-excluded surface) was built using this framework and implemented across four platforms and libraries. We applied a set of metrics borrowed from computer vision to assess the accuracy and efficiency of our model. Its performance was validated over three distinct data sets, demonstrating that our model is insensitive to grid spacing, suggesting further potential improvements in efficiency without compromising accuracy.

**Figure 1:**
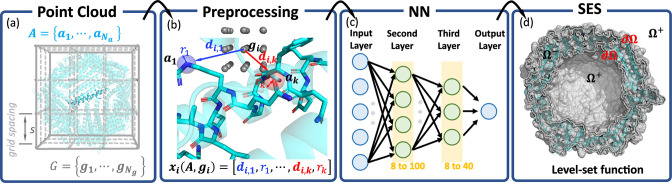
Schematic illustration of the workflow: (a) point cloud setup, digital representation of a specific molecule; (b) preprocessing the point cloud to fulfill the invariance of surface with respect to the translation and rotation of the molecule and insensitivity to the predefined grid spacing; (c) construction of neural network (NN) for fitting the level-set function; (d) construction of SES surface using the level-set function. Here, the point cloud presentation of a helical protein is illustrated with an artificially coarse grid. One specific helix of the protein is highlighted for subsequent processing.

In this study, we utilized three distinct data sets. For model training, we employed a set of 573 proteins derived from the AMBER PBSA benchmark suite. This data set comprised biomolecules with 377 to 8254 atoms, offering a diverse array of geometries. The training and benchmark data for our model was derived from the AMBER/PBSA surface builder, which was tailored for the geometry-based SES, herein denoted as “classical SES”.^24^ This approach followed the fundamental principles articulated by You and Bashford^13^ and Rocchia et al.^21^ We stratified this data set, allocating 20% for testing, with the remaining 80% partitioned into training and validation data sets. To evaluate our model’s transferability, we compiled a data set of 364 biomolecular structures of nucleic acids, given their distinct functional and rigidity characteristics compared to proteins. In addition, we assembled a data set of 622 protein complex structures, which are significantly larger than single proteins, to test the model’s scalability. A detailed summary of these three data sets can be found in the Supporting Information (Section S1).

The level-set function has been extensively used for representing the SES of a molecule due to its convenience.^19,24^ Within this framework, the entire surface is discretized into a three-dimensional (3D) grid space ***G*** = {**g**_1_,···,**g**_*N*__g_} containing *N*_*g*_ grid points, where  signifies the coordinate of the *i*-th point in the 3D space. The distance between two nearest neighbor points is a constant value *s* ∈ (0, 1] (also referred to as the grid spacing, Figure 1a). The sign of level-set values (*y*_*i*_) indicates whether a grid point **g**_*i*_ is positioned outside-of-boundary or inside-of-boundary^26^ (as illustrated in Figure 1d),
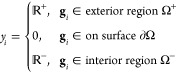
1where Ω^+^ represents the exterior surface region and Ω^–^ corresponds to the interior region,  and  are corresponding positive and negative level-set values, respectively. The sign transition between the inside and outside is made at point ∂Ω, referred to as the surface. The surface, therefore, can be ascertained by identifying the phase of sign alteration.

The concept of point cloud was introduced here for the representation of 3D molecular coordinates,^36−38^ where it consists of a set of data points with each point denoting the specific position of atoms in molecular systems. For a molecule containing *N*_*a*_ atoms, it can be expressed in digital format (**A**)
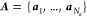
2where  represents point located in the center of atom *i* within a specified 3D space.

With the digital format of 3D molecules and grid space available, the surface generation problem simplifies to mapping the 3D molecules and grid space into the level-set function. The objective is to identify function ϕ as defined below:
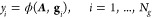
3

Upon correctly generating level-set values at grid points, a continuous surface representation can be constructed from discretely defined level-set values using an appropriate interpolation function. This serves as a key mechanism in various scientific processes, particularly those relying on grid mapping.^39^ Quadratic or trilinear interpolation functions are typically employed for interpolation.^40^ These functions are instrumental in the construction of surface ∂Ω. Thus, the molecule surface ∂Ω can be defined via the interpolation function by identifying the position where the corresponding level-set value equals zero:
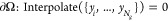
4where Interpolate(•) is the interpolation function, *y*_*i*_ is the level-set value of grid point **g**_*i*_. As a result, resolving the molecule surface construction problem is essentially reduced to modeling the level-set function ϕ(•). Accurate estimation of this function allows for a precise representation of any type of surface of any given molecule.

Neural networks have demonstrated versatility and aptitude in fitting functions of any form, provided there is a sufficient amount of data. In this work, a neural network was utilized to estimate the function ϕ(•) (cf. eq 3). Although the general idea is straightforward, two significant challenges need to be emphasized: (i) the need for surface invariance with respect to the translation and rotation of a particular molecule; (ii) the requirement for insensitivity to the predefined grid spacing (*s*).

In order to achieve surface invariance, we adopted the relative distance as the feature of our model. Moreover, the coordinates of the nearest-*k* atoms {**a**_1_, ..., **a**_*k*_} around the queried grid point **g**_*i*_, along with their corresponding radii, were chosen as the surrounding environments. This was done to improve the representation and decrease the grid-spcaing dependency. Additionally, this makes our method more suitable for adapting changes from local modifications, such as rotamer shifts in residues, given a previously built surface. Such features could further shorten the time consumption in the surface generation and are distinct from other methods that necessitate a complete rebuild. The mathematical expression for a specific grid point **g**_*i*_ is formulated as follows:
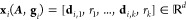
5where *r*_*i*_ denotes the radius of the nearby atom **a**_*i*_, **d**_*i*,*j*_ designates the relative distance vector (**d**_*i*,*j*_ = **a**_*j*_ – **g**_*i*_) established between target grid point **g**_*i*_ and the nearby atom **a**_*j*_, and *d* is the dimension of the feature vector **x**_*i*_ (Figure 1b). A statistical analysis was conducted on the number of nearby atoms in our experiment, and the maximum value (*k*) did not exceed 24 (Figure S2). Hence, 24 nearby atoms around each grid point were chosen, and the final dimension of the feature vector (*d*) for training and inference was set to be 96.

Utilizing the aforementioned input features (**x**_*i*_) and objective (Figure 1d), the task can be generalized as below,
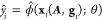
6where θ symbolizes the trainable parameters of the neural network. The aim of the training objective function is to minimize the disparity between the ground-truth level-set value *y*_*i*_ that was directly extracted from the AMBER PBSA benchmark suite and the prediction 
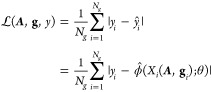
7Since the model is fully differentiable, a standard gradient descent algorithm^41^ can be employed to update the model parameter θ to minimize the given objective function:
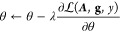
8where  represents the preset learning rate parameter. For more comprehensive details on the loss function, optimization algorithm, learning rate, and neural network construction, please refer to Figure 1c and Supporting Information. (Section S2 and Section S3)

In order to robustly evaluate the performance of the model, this study employs metrics designed to effectively quantify the degree of accuracy between the predicted and actual surfaces. The selected metrics are the coefficient of determination^42−45^ (*R*^2^) and the Mean Absolute Error^46−49^ (MAE), which assess the level-set value prediction performance. In order to further ascertain the accuracy of surface construction, we employ the Chamfer Distance^50,51^ (CD) and the *F*-score,^52,53^ both commonly used in 3D structure research.^54,55^

Chamfer distance (CD) is a metric for evaluating the similarity between two point sets.^56,57^ It is computed by aggregating the Euclidean distances between nearest neighbor correspondences from two point clouds. In our setting, we have the predicted boundary point set ∂Ω̂ and the ground truth point set ∂Ω, therefore, for each point *x* ∈ ∂Ω̂, *y* ∈ ∂Ω, the Chamfer Distance is defined as
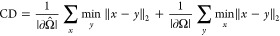
9where a lower distance value indicates a more accurate surface estimation.

The *F*-score is calculated between two sets of points, with a hit denoted by the existence of two points within a defined radius *r* of each other.^56^ The *F*-score is formulated as
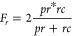
10where *pr* denotes the precision score, and *rc* signifies the recall value. The *F*-score can be interpreted as a harmonic mean of precision and recall with the optimal *F*-score value being 1 and the worst value being 0.

In this work, we employed a three-layer neural network. Within this architecture, the size of the second layer is selected from the set {8, 16, 32, 64, 96, 100}, while the size of the third layer is chosen within the set {8, 16, 32, 40} (Figure 1c). An exhaustive grid search was conducted to optimize the balance between accuracy and efficiency. As demonstrated in Figure 2, both MAE and *R*^2^ achieve better results when increasing the number of nodes within each layer. For instance, by fixing the number of nodes in the second layer to 64 and increasing the number of nodes in the third layer from 8 to 40, MAE decreases from 0.103 to 0.100 and *R*^2^ increases from 0.943 to 0.945. However, solely increasing the nodes can substantially increase the computational burden and may lead to overfitting.^58^ Taking into account both parameter size and model performance, we found the combination of 64 and 32 appears to be a good choice. All models in the subsequent section were trained using this combination.

**Figure 2:**
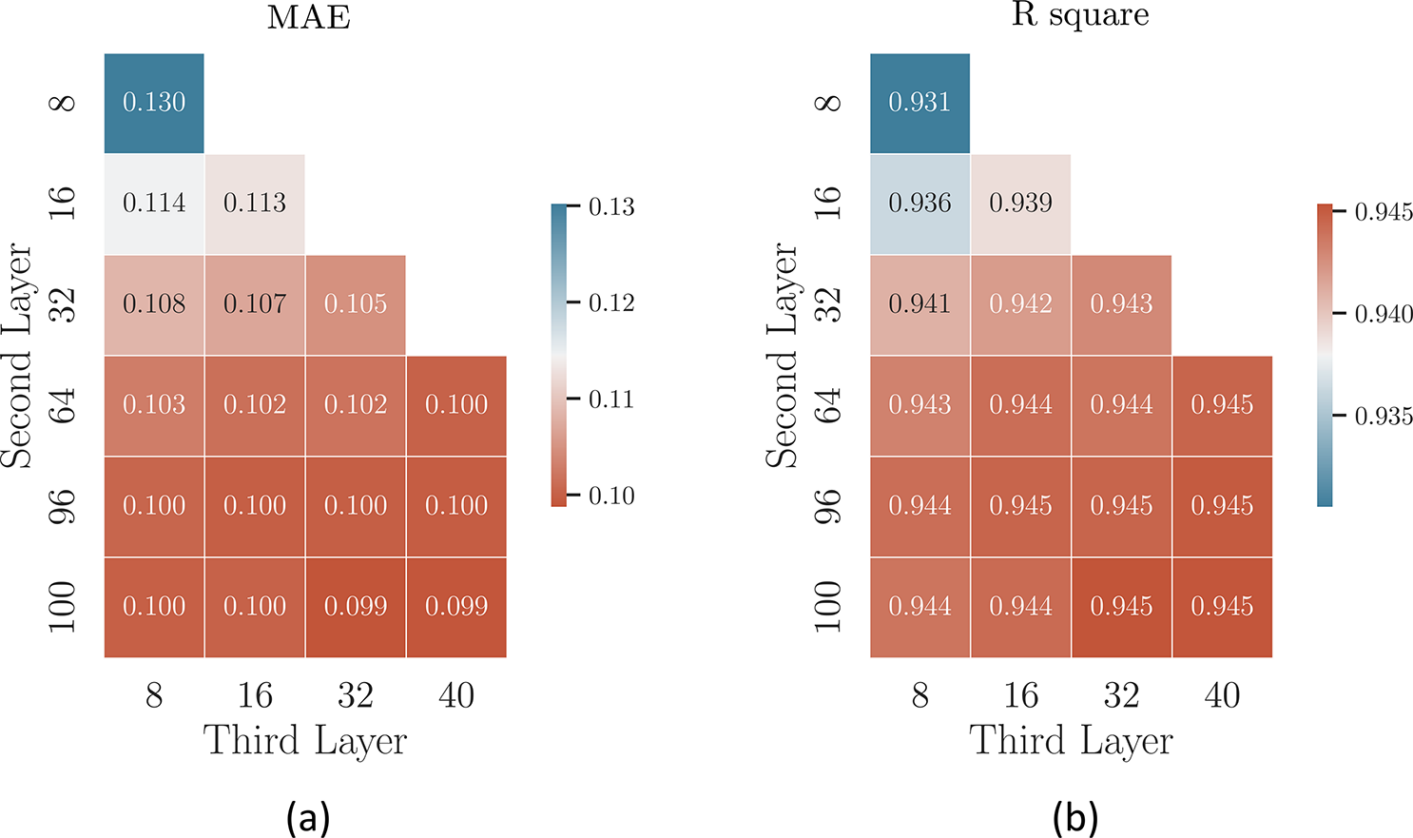
Estimation of model performance in the term of (a) MAE and (b) *R*^2^ with different architectures, respectively. Performances are also color-coded, where higher performances are shown as warmer colors, as indicated by the respective scales.

To assess the accuracy of the GENIUSES model, the difference between the surfaces predicted by our model and those predicted by classical approaches offers a direct measure. As shown in Figure 3 a–c, superimposed surfaces predicted by the GENIUSES (blue) and the classical SES (red) are presented with respect to various shapes and conformations. From these superimposed surfaces, it can be observed that there are no noticeable visual discrepancies, since the points (blue) generated by the GENIUSES model closely coincide with those from the classical SES (red). This observation aligns with the metrics calculated based on Chamfer Distance (CD), and *F*-score (inserted in the top of Figure 3a–c).

**Figure 3:**
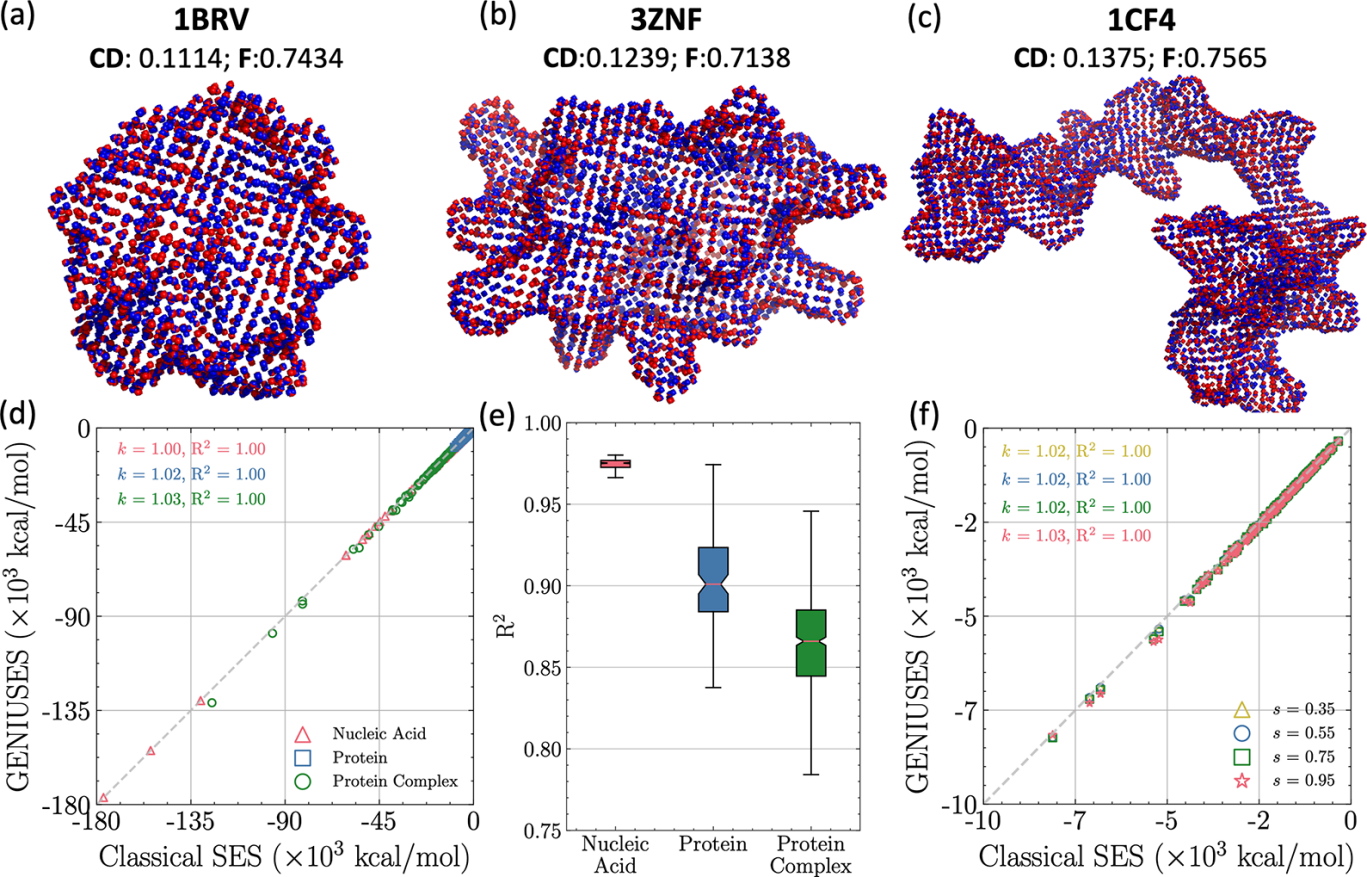
Model accuracy and robustness with different test systems and grid spacings. (a–c) Superimposed surface generated by GENIUSES (blue) and classical SES (red) for representative molecular structures, corresponding PDB ID’s and metric values are also shown. (d) Comparison of PB reaction field energies across three different data sets with classical SES surface and GENIUSES surface. Here grid spacing is set to be 0.35 Å. (e) *R*-squared values of GENIUSES for the three data sets. (f) Comparison of PB reaction field energies between the surface predicted by GENIUSES with different grid spacings (*s* = 0.35, 0.55, 0.75, 0.95 Å) and classical SES surface with 0.35 Å grid spacing.

Additionally, we compared our model to MLSES, a relevant work in the context of machine learning for SES modeling.^27^ As outlined in Table 1, the GENIUSES model outperforms the MLSES model significantly. The GENIUSES model achieved a markedly lower CD score (CD = 0.1309) compared to the MLSES model (CD = 0.2312). Given that the CD represents the Euclidean distances between the closest corresponding points of two point clouds on the Å scale, a deviation of 0.2312 is considerable, indicating a high degree of error. However, the GENIUSES model reduces this error by 43.38%. Moreover, the GENIUSES model surpasses the MLSES model in terms of *F*-score (*F* = 0.9224 vs *F* = 0.8421). The GENIUSES model also attains an *R*^2^ value of 0.9438 and an MAE of 0.1019. As the MLSES model is classification-based, it does not provide *R*^2^ or MAE values. Besides, its classification task introduces sensitivity in surface construction.

Upon investigating less accurate cases, we identified that such errors mainly come from the interior region where self-intersection could occur between internal cavity and an accessible region^17^ (Figure S4). Such inaccuracy mainly comes from the imbalance between data distribution of the exterior and interior (Figure S1). Further details regarding this comparison can be found in the Supporting Information, Section S4.

**Table 1:** Quantitative Analysis of Model Performance with CD, *F* (with Autodetermined Radius Value), *R*^2^, and MAE

Model	CD(*↓*)	*F*(*↑*)	*R*^2^(*↑*)	MAE(*↓*)
MLSES	0.2312	0.8421	–	–
GENIUSES	**0.1309**	**0.9224**	0.9438	0.1019

In a more realistic scenario, the estimation of Poisson–Boltzmann reaction field energies utilizing predicted surface was conducted and compared with energies calculated using classical SES when both utilized a grid spacing of 0.35 Å. As shown in Figure 3d, an excellent performance is demonstrated in PB energy calculations. In the case of the protein data set (blue square), which contains 573 molecules, *R*^2^ of fitted function reached 1.00 and the corresponding slope is 1.02 showing a slight deviation from 1.00. These results collectively confirm the accuracy of our model in terms of surface coincidence and PB energy calculations.

The robustness of our model is initially demonstrated through its transferability. The GENIUSES model, trained exclusively on a protein data set, is subsequently applied directly to surface generation for both nucleic acid and protein complex data sets where the structural flexibility and pattern are quite different from training ones. Among these data sets, the nucleic acid data set encompasses a more diverse range of conformations, while the protein complex data set comprises more intricate structures (Section S1). Visual distinctions between surfaces generated by our approach and conventional methods are illustrated in Supporting Information, Figure S5. Estimated PB energies across the nucleic acid and protein complex data sets are presented in Figure 3d. It can be concluded from these results that our model maintains an exceptional performance as evidenced by all *R*^2^ is 1.00. Only a minor decrease in slope is observed for the protein complex data set, attributed mainly to its intricate structure. The same conclusion can be drawn from *R*^2^. As illustrated in Figure 3e, the GENIUSES model displays exceptional proficiency on the protein validation data set, achieving an impressive accuracy exceeding 90%. For the nucleic acid data set, the *R*^2^ value at Q1 is approximately 0.97, and at Q3 it is around 0.99. The mean *R*^2^ value, lying at 0.98, suggests an overall average performance for nucleic acids that exceeds 98%. Similarly, the overall average performance for protein complex is above 87.5%. The same trend applies to MAE, as shown in Supporting Information, Figure S6.

We conducted an analysis on the accuracy of the surface generation across a variety of grid spacings. A crucial observation from Table 2 is that the accuracy of MLSES model is heavily dependent on the grid spacing. For example, the value of the CD score escalates from 0.1059 to 0.2312, indicating an error rate increase of 118% when *s* rises from 0.35 to 0.95. In stark contrast, the GENIUSES model demonstrates remarkable robustness against variations in grid spacing, as evidenced by the slight change in CD score (an increase from 0.1035 to 0.1309, corresponding to a modest degradation of 26%) when *s* progresses from 0.35 to 0.95. This pattern is also consistent with the *F*-score. As *s* expands from 0.35 to 0.95, the MLSES *F*-score plunges from 0.7311 to 0.3718, marking an error rate increase of 49%. Meanwhile, the GENIUSES model demonstrates a modest *F*-score decrease from 0.7360 to 0.6833, signifying only a 7% degradation. A detailed examination of Table 2 reveals that at a grid spacing *s* = 0.95, the GENIUSES model achieves superior performance with only 30,000 data points, compared to the MLSES model at grid spacing *s* = 0.55 that requires 150,000 data points. The premise of fewer data points ensures the efficiency of our model, which is expounded upon in the following section.

**Table 2:** Quantitative Analysis of Model Performance with CD and *F* at Different Grid Spacings (*s*)^a^

Model	*s* (Å)	*N*_g_	CD(*↓*)	*F*_0.15_(*↑*)
MLSES	0.35	560K	0.1059	0.7311
	0.55	150K	0.1459	0.5697
	0.75	60K	0.1882	0.4535
	0.95	30K	0.2312	0.3718
GENIUSES	0.35	560K	**0.1035**	**0.7360**
	0.55	150K	**0.1145**	**0.7116**
	0.75	60K	**0.1230**	**0.6978**
	0.95	30K	**0.1309**	**0.6833**

a The radius (*r*) required for the *F*-score calculation was kept constant at 0.15 Å. *N*_g_ is the number of total grid points for a given target molecule under current grid spacing.

The PB energy calculations performed over the protein data set using different grid spacings also manifest the robustness of our model. As depicted in Figure 3f, no significant discrepancies were observed in the energy estimation when employing different grid spacings. The correlations between the energies using the predicted surface of varying different grid spacings (*s* = 0.35, 0.55, 0.75, and 0.95 Å) and those derived from the classical SES surface with a grid spacing of 0.35 Å all remain 1.00, and the slopes of the fitted linear line approach 1.00. These results validate the robustness of our model concerning surface generation and subsequent applications.

At the heart of GENIUSES lies the treatment of matrix multiplication, which enables its efficient implementation across various platforms (CPU and GPU) or libraries (Torch and CUDA). Here, to validate its efficiency and evaluate its performance across these platforms and libraries, we accommodated four distinct kernel configurations (Fortran, Torch CPU, Torch CUDA, and CUDA) to meet different utilization scenarios. The detailed implementation of this method across various libraries and platforms is discussed in Section S6.1 in the Supporting Information.

Using the build-in classical SES procedure in AMBER/PBSA as a benchmark, we also compared the time consumption among widely used classical implementations of SES methods, namely EDTSurf^18^ and NanoShaper,^17^ as well as the machine-learned method MLSES with our model implemented across various platforms and libraries. For a fair comparison, we disabled the printing of intermediate grid points information in both AMBER/PBSA and GENIUSES, only retaining the printing of surface information, consistent with other SES programs. As shown in Figure 4a, with increasing number of atoms, all methods tested in this work show a consistent trend. This trend coincides with our intuition that the task of surface construction is proportional to the number of atoms. For systems containing fewer than 2000 atoms, regardless of the platform or library utilized, our method (solid circle) significantly outperforms the classical SES (blue “X”) and MLSES (pink “X”). When the atom count exceeds 2000, most implementations of the method still outperforms the classical SES (blue “X”) and MLSES (pink “X”), except that implemented with the Kernel Fortran on CPU platform (green solid circle), which exhibits a marginally slower speed than MLSES (pink “X”).

**Figure 4:**
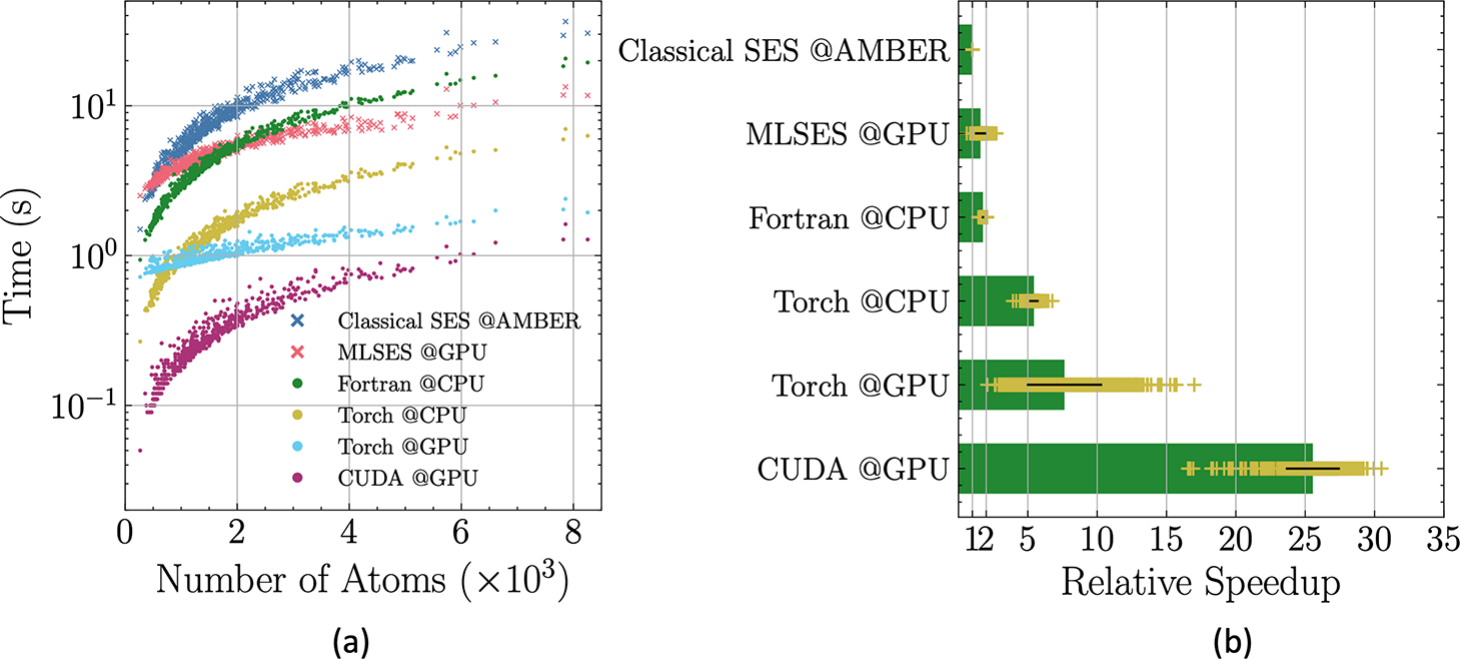
Comparative analysis of performance with average run time and relative speedup for the protein data set. (a) Run time as a function of number of atoms using different methods and GENIUSES implemented on different platforms. GENIUSES related methods are all represented in solid circles, while Classical SES and MLSES are in the style of “X”. (b) Relative speedup with respect to the Classical SES.

The efficiency of our method is further quantified by the relative speed compared with the classical SES fromAMBER/PBSA, as shown in Figure 4b and Table 3. The relative speedup average is calculated by taking the average of relative speedup on the each molecule between the different SES programs and the benchmark. From this analysis, we can safely conclude that 26-fold speedup of our model with respect to the classical SES could be achieved over the protein data set when utilizing Kernel CUDA. (Table 3) Even with a CPU implementation (LibTorch GENIUSES), our model still exhibits a 5-fold speed-up compared to the classical SES. These results were consistently observed over the other two data sets, one of which includes much larger protein complex structures (Figure S7). Comparison was further conducted over widely used classical implementations of SES methods, specifically EDTSurf and NanoShaper. As detailed in Table 3, the EDTSurf method exhibits a slightly slower surface construction rate compared to the benchmark method with a speedup of 0.37 and 0.66 over nucleic acid and protein data set, respectively. For NanoShaper, an 8-fold speedup was achieved over protein data set when utilizing only one thread. Such a speedup increases to around 19 when applying 32 threads. Further increasing threads to 64 does not significantly accelerate its speed. The surface generation speed is highly dependent on the molecular size.^17^ A comprehensive comparison over large-scale protein complexes was conducted and listed in Table 3. Both EDTSurf and NanoShaper outperform the classical SES implemented in AMBER, the speedup for EDTSurf is 2.15 and 18.16 for NanoShaper when utilizing 64 threads. For our method, its scalability was further demonstrated by a remarkable relative speedup of 33.28 over data set protein complex when utilizing Kernel CUDA. Given these findings, we further envision that the method could be used in the process of drug screening where computational speed is a critical factor. In a concerted effort to benefit the broader research community, the efficient implementation of our proposed method has been integrated into the widely used molecular modeling software package, AMBER.^59^

**Table 3:** Comparative Analysis of Performance with Average Run Time and Relative Speedup for the Nucleic Acids, Protein, and Protein Complex Data Sets among Different SES Programs^a^

			nucleic acid		protein		protein complex	
Methods	CPU	GPU	avg. time (std.) (s)	rel. speedup	avg. time (std.) (s)	rel. speedup	avg. time (std.) (s)	rel. speedup
Classical SES@AMBER	1	0	4.45 (3.32)	1.00	8.30 (4.77)	1.00	35.07 (42.94)	1.00
GENIUSES Torch@CPU	1	0	0.88 (0.62)	5.02	1.54 (0.92)	5.44	6.87 (8.09)	4.87
GENIUSES Torch@GPU	1	1	1.16 (0.40)	3.61	1.03 (0.21)	7.64	1.67 (1.17)	17.52
GENIUSES CUDA@GPU	1	1	0.19 (0.15)	23.38	0.33 (0.20)	25.55	1.00 (1.18)	33.28
EDTSurf	1	0	14.69 (4.68)	0.37	14.09 (4.42)	0.66	15.32 (6.59)	2.15
NanoShaper 1 Thread	1	0	0.68 (0.45)	6.42	1.02 (0.50)	7.97	5.27 (8.35)	7.74
NanoShaper 32 Threads	32	0	0.27 (0.21)	16.98	0.43 (0.25)	19.30	2.43 (3.65)	15.86
NanoShaper 64 Threads	64	0	0.27 (0.21)	16.81	0.44 (0.25)	19.22	2.05 (2.89)	18.16

a All speedups are with respect to the Classical SES from AMBER/PBSA.

In this work, we developed a universal framework for surface construction that combines point cloud and neural networks, effectively ensuring efficiency and accuracy. Notably, this framework can be effortlessly adapted across different platforms and libraries, including CPU, GPU, and related libraries. To demonstrate its performance, we deployed this framework in a model (GENIUSES) for the generation of solvent-excluded surfaces (SES), due to its complexity. In terms of accuracy, our model can achieve ∼95% fidelity compared with the classical SES method implemented in AMBER. The consistency between the PB energies computed with the GENIUSES surface and those with the classical SES further validates the accuracy of our model. In terms of efficiency, the GENIUSES CUDA implementation on GPU can yield a speedup about 26 times over the classical SES of AMBER which is limited to the CPU platforms. Notably, in large-scale systems, GENIUSES delivers an even more remarkable speedup of 33 times. Moreover, our analysis further indicates that the model is robust with respect to changes in grid spacing and is scalable to larger systems without much loss of accuracy or efficiency. For the benefit of the broader scientific community, we integrated our model into the popular AMBER platform and made it fully open-source. We believe that this model will serve as a powerful and efficient tool for large-scale molecular surface analysis. Despite these achievements, there is still room for improvement, particularly in the treatment of inner cavities, which will be the focus of our future development.

## Supporting Information Available

Information on data sets, model detailed structures, training details, baseline comparisons, surface construction performance, and inference performance (PDF)
Transparent Peer Review report available (PDF)
