# Supplementary material for: Grid-Robust Efficient Neural Interface Model for Universal Molecule Surface Construction from Point Clouds
Source: J Phys Chem Lett. 2023 Oct 2;14(40):9034–41. doi: 10.1021/acs.jpclett.3c02176 (PMC10577766; doi:10.1021/acs.jpclett.3c02176)
Supplement: Supplementary file 1 — jz3c02176_si_001.pdf [file jz3c02176_si_001.pdf]

Grid-robust Efficient Neural Interface Model for Universal Molecule Surface Construction from Point Clouds

Yongxian Wu,[†] Haixin Wei,[‡] Qiang Zhu,[*,†] and Ray Luo[*,†]

†*Department of Chemical and Biomolecular Engineering, Molecular Biology and Biochemistry, Materials Science and Engineering, and Biomedical Engineering, University of California, Irvine, California 92697, United States*

‡*Department of Chemistry and Biochemistry, University of California, San Diego, California 92093, United States*

E-mail: qiangz11@uci.edu; rluo@uci.edu

Contents

S1 Datasets **S3**

S1.1 Benchmark Dataset S3

S1.2 Cross-system Dataset S3

S1.3 Interior Points Distribution S4

S2 Model Detailed Structures **S4**

S2.1 Evaluation Metrics S5

S3 Training Details **S6**

S3.1 Optimization Settings S6

S3.2 Training Process S7

S4 Baseline Comparisons **S8**

S4.1 Compare with Previous Work S8

S5 Performance Estimation **S10**

S5.1 Surface Visualization S10

S5.2 Cross-system validation S11

S6 Inference Performance **S12**

S6.1 Implementation Details S12

S6.2 Inference Time S13

S7 Comparison with the NanoShaper based DelPhi **S13**

References **S14**

S1 Datasets

S1.1 Benchmark Dataset

Training Dataset The training data for our model was obtained from the AMBER[1] PBSA benchmark suite, which is a comprehensive resource consisting of 573 biomolecular protein structures. The atomic counts of these biomolecules range from 377 to 8254, providing a diverse set of geometries.

We generated the training data using a custom PBSA program. This program utilized default atomic cavity radii, extracted from topology files, and a solvent probe radius set to 1.4 Å. Data collection for benchmarking was performed at grid spacings of 0.35 Å and 0.55 Å, covering the entire dataset. For data augmentation, additional boundary-specific data were gathered at a grid spacing of 0.55 Å. All other parameters were maintained at their default values as specified in the PBSA module of the AMBER 22 package.

Through this meticulous process, we acquired a dataset comprising 248,380,694 data points in total. To stratify the dataset, 20% of the data was allocated for testing purposes, while the remaining 80% was further divided into training and evaluation datasets. We used a test size of 0.1 and a consistent random seed for reliable reproducibility during the train-test split.

S1.2 Cross-system Dataset

To evaluate the generalization capabilities of our model, we employed additional unseen datasets, consisting of a nucleic acid dataset and a protein complex dataset.

Nucleic Acid The nucleic acid dataset comprises a total of 364 biomolecular structures from the AMBER benchmark suite. These biomolecules vary in size, with the number of atoms ranging from 250 to 5569, and exhibit diverse geometries.

Protein Complex The protein complex dataset includes 622 protein complex structures from the AMBER benchmark suite. These biomolecules also demonstrate a wide range of geometries, with atom counts varying from 660 to 142,324.

S1.3 Interior Points Distribution

We investigate the distribution of interior grid points within the complete set of grid samples from the protein dataset. As depicted in Figure S1, the average proportion of interior points is consistently below 3.10% throughout the dataset.

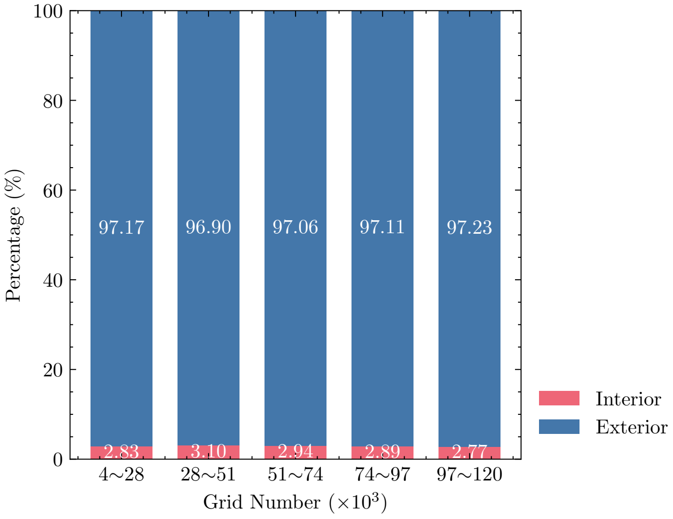

Figure S1: Percentage of interior points within the protein dataset.

S2 Model Detailed Structures

The design of our neural network model prioritizes efficiency and compatibility with computational hardware. Specifically, we utilize a three-layer feed-forward network (FFN) with the architecture 96-64-32-1. This choice aims to accommodate a vicinity of 24 atoms, a number determined by examining our dataset, which revealed that most atoms have 24 neighbors, as depicted in Figure S2.

The architecture was further optimized, taking into account the hardware characteristics

of GPUs and CPUs, which exhibit a preference for base-2 systems. GPUs, in particular, favor memory sizes aligned to multiples of 8, reducing memory conflicts and promoting efficient computation. Therefore, we deliberately selected node numbers in multiples of 8 for the second and third layers, thereby enhancing computational efficiency while mitigating bank conflicts.

The activation function incorporated within the network is the Rectified Linear Unit (ReLU). In terms of data preparation, we repositioned the original labels, which initially ranged from [-1.5, 1.5], (the value highly depends on the solvent probe radius) to fit the interval [0, 3]. This adjustment ensures an effective input representation for the network.

S2.1 Evaluation Metrics

The coefficient of determination (R^2) and the Mean Absolute Error (MAE) are two commonly utilized performance metrics. Additionally, to assess the surface estimation accuracy, we adopt the Chamfer Distance (CD) and the F score to evaluate the boundary accuracy.

Coefficient of Determination The coefficient of determination, also known as R^2, quantifies the proportion of the variance in the dependent variable that can be predicted from the independent variables.[2] In our setting, the R^2 score can be computed as follows:

$$\mathrm{R}^2 = 1 - \frac{\sum_{i=1}^{n}(y_i - \hat{y}_i)^2}{\sum_{i=1}^{n}(y_i - \bar{y})^2} \tag{1}$$

where n is the number of samples, y_i is the truth level-set value that directly extracted from the AMBER PBSA benchmark suite, $\hat{y}_i$ is the predicted value by our model, and $\bar{y}$ is the mean of the observed values. An R^2 score of 1 indicates perfect prediction, while a score of 0 suggests that the model does not improve predictions over using only the mean of the target variable.

Mean Absolute Error The Mean Absolute Error (MAE) is another popular metric used in model evaluation. It is defined as the average of the absolute differences between the predicted and observed values.[3] In our setting, the MAE can be calculated as:

$$\mathrm{MAE} = \frac{1}{n}\sum_{i=1}^{n}|\hat{y}_i - y_i| \tag{2}$$

where n is the number of samples, y_i is the truth level-set value that directly extracted from the AMBER PBSA benchmark suite, and $\hat{y}_i$ is the predicted value by our model. MAE offers a straightforward interpretation, representing the average magnitude of the errors made by the model in its predictions, regardless of their direction. Lower MAE values indicate better predictive accuracy, with an MAE of 0 signifying a perfect predictor.

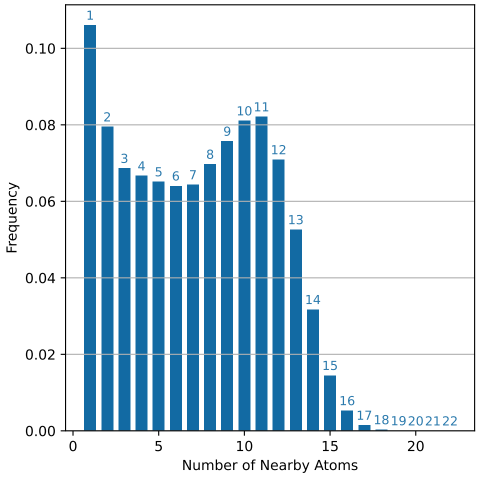

Figure S2: Frequency distribution of the number of nearby atoms at a certain grid point.

S3 Training Details

S3.1 Optimization Settings

We implemented the neural network model using the PyTorch framework.[4] The model was trained for 10 epochs, consisting of 2,417,000 steps to achieve convergence. The learning rate

was set to $1e^{-4}$, and the batch size was set to 64. The training process was executed on a single GPU card (NVIDIA GeForce RTX 3090) and took approximately 20 hours.

S3.2 Training Process

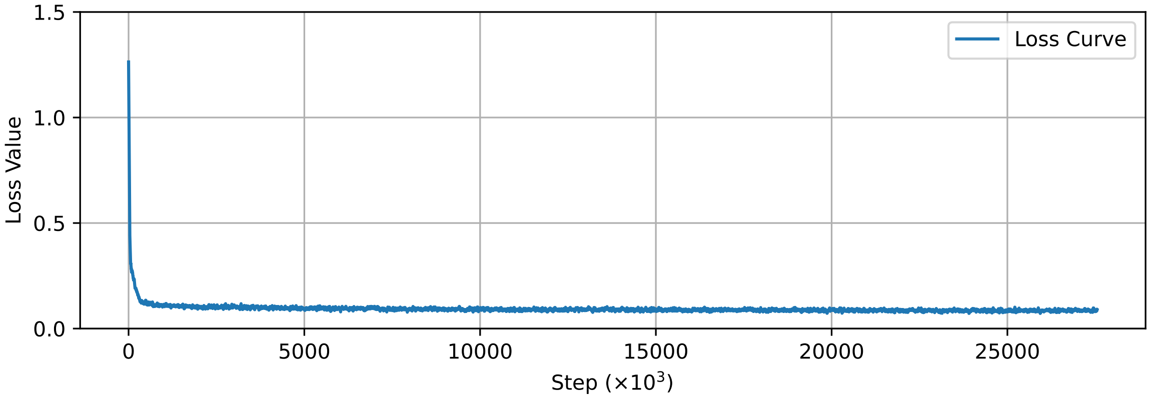

Figure S3: Loss curve during the training stage.

S4 Baseline Comparisons

S4.1 Compare with Previous Work

Classical Implementation of SES methods Traditional methodologies primarily focus on the physical interactions among atoms within a given molecule. Among these, the classical implementation of solvent-excluded surface (SES) approaches in AMBER/PBSA ,[5–7] EDTSurf[8] and NanoShaper[9] stand out as well-researched and widely used strateges in computational chemistry and molecular modeling for representing a molecule's surface in relation to its interaction with a solvent. The build-in classical implementation of SES method in AMBER/PBSA is specifically designed for geometry-based SES the geometry-based SES, referred to as "classical SES"[10] in this paper, following the basic ideas of Connolly's surface definition[6] and the foundational principles outlined by You and Bashford[11] and Rocchia et al.[12]. The SES is essentially a smooth, continuous boundary that encapsulates the volume inaccessible to solvent molecules. In the classical implementation of SES methods, ϕ is determined by rolling a probe sphere, simulating the solvent molecule, over the van der Waals surface of the solute molecule. The radius of this probe sphere is typically approximated to the size of a water molecule, around 1.4 Å.

Despite its ability to provide accurate surface estimations, the classical implementation of SES methods can be computationally demanding and time-intensive, especially for large systems or when high-resolution surface representations are required. Additionally, parallelizing the classical SES method poses intrinsic challenges, limiting its effective utilization of the GPU's escalating computational capabilities, which have demonstrated substantial potential in high-performance computing.

MLSES MLSES is a recently proposed SES generation method which also utilized a neural network framework to predict grid and subsequently estimate the SES surface.[13] Nevertheless, the MLSES method succumbed to the pitfalls of classification and overlooked the critical

issue of grid-scale robustness. This is an area in which our proposed methodology demonstrates significant prowess. As the sections in the main text reveal, their model exhibited sensitivity to the grid scale, significantly affecting the accuracy of grid mapping and imposing limitations on the final SES depiction. Furthermore, the computational speed improvements offered by their method were limited, falling short of the anticipated efficiency gains that are typically expected of machine learning applications.

S5 Performance Estimation

S5.1 Surface Visualization

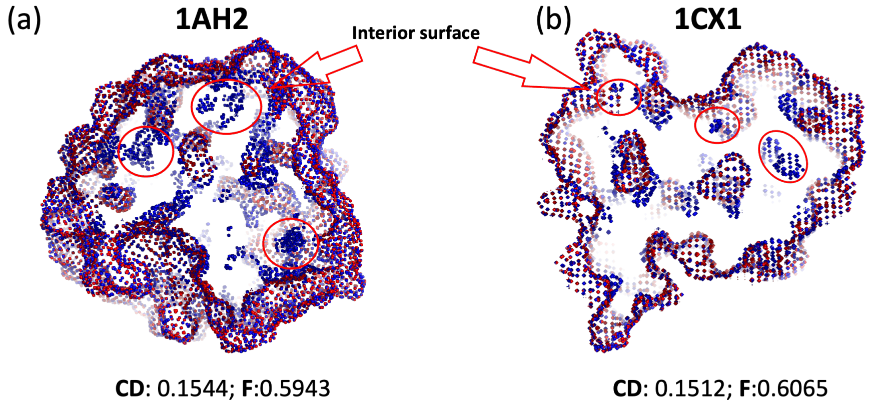

Figure S4: Illustration on the divergence between the predicted and original surface.

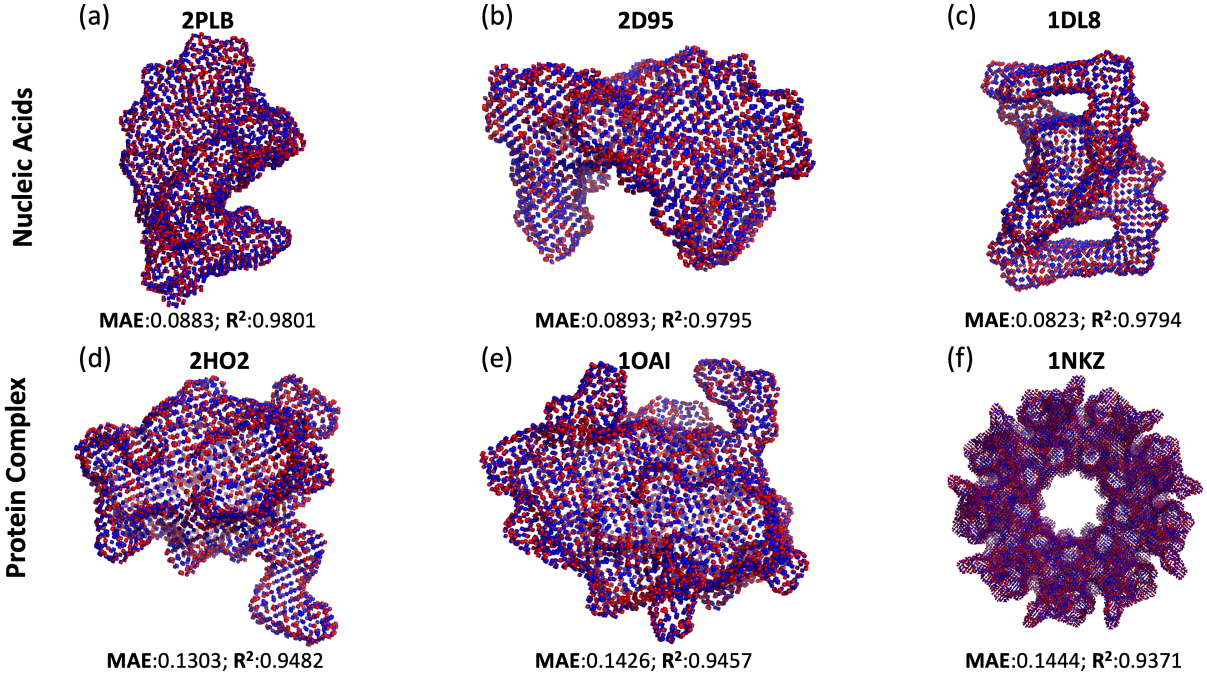

Figure S5: Model performance validated on dataset nucleic acids (a-c) and protein complex (d-f). Together shown are their corresponding PDB ID and metric values (MAE and R^2) Surface generated by classical SES and our GENIUSES are colored in red and blue, respectively.

S5.2 Cross-system validation

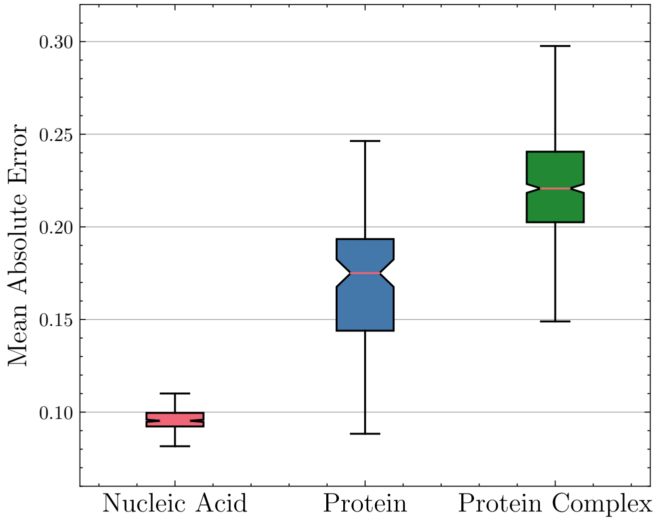

Figure S6: MAE values of GENIUSES validated on three different datasets.

S6 Inference Performance

S6.1 Implementation Details

We have successfully integrated the GENIUSES model into AMBER,[14] leveraging the capabilities of LibTorch, a C++ runtime library developed by the PyTorch team.[4] For comprehensive details on utilizing our implemented method, we direct readers to consult the AMBER 2023 Reference Manual[1]. The speed tests reported in the paper for GPU implementation were measured on CUDA 11.6.

Intergration of LibTorch This library enables flexible tensor computations and dynamic deep neural network modeling, allowing developers to deploy trained neural network models from PyTorch directly to C++ platforms without the need for a Python interpreter. In our AMBER implementation, we incorporate LibTorch for inference using the GENIUSES model in a pure C++ runtime. This approach results in satisfactory performance acceleration on both CPU and GPU environments, eliminating the necessity for intricate, hand-crafted optimizations.

Moreover, LibTorch provides general-purpose, high-performance numerical computing capabilities through tensor abstraction, which is advantageous for AMBER developers aiming to design efficient numerical algorithms and integrate custom machine learning models into the software.

There are two methods for enabling LibTorch in AMBER: built-in mode and user-installed mode. In the built-in mode, LibTorch is installed automatically during the AMBER compilation process. Version 1.12.1 supports computing runtimes on CPU, CUDA 11.6, CUDA 11.3, and CUDA 10.3. The specific runtime is determined by the user's configuration, such as the CUDA setting state.

[1] `http://ambermd.org/doc12/Amber23.pdf`

Custom CUDA Implementation To reduce the overhead caused by the LibTorch framework, we further develop custom CUDA kernel for our GENIUSES inference model. The kernel can perform inference with high performance without relying on the LibTorch framework. We test it on CUDA 11.3 and CUDA 11.6.

S6.2 Inference Time

For a fair comparison, we disabled the printing of intermediate grid points information in both AMBER/PBSA and GENIUSES, only retaining the printing of surface information, consistent with other SES programs.

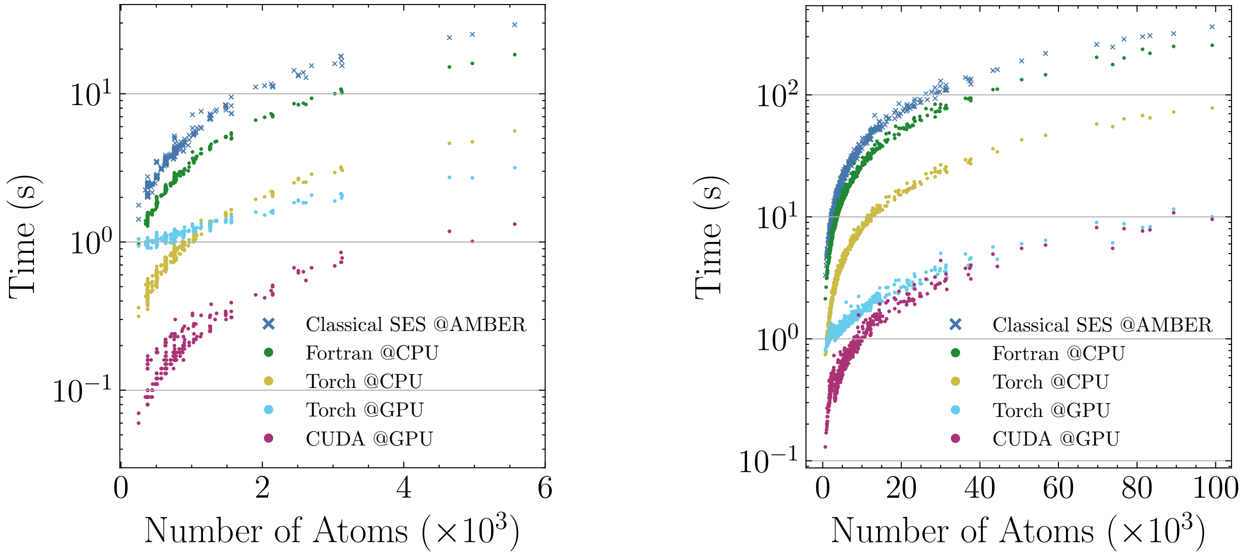

(a) nucleic acid

(b) protein complex

Figure S7: Average performance running time analysis on (a) the nucleic acid and (b) the protein complex system.

S7 Comparison with the NanoShaper based DelPhi

To further understand the benefit of our GENIUSES method to the AMBER PBSA, we conduct a timing experiment on comparing the inference performance of our AMBER PB solver (PBSA) against another state-of-the-art PB solver, DelPhi, where NanoShaper is proposed

to speed up the DelPhi program by accelerating the molecule surface building process. For the NanoShaper, we compile the latest release version 0.7 and follow the instruction provided in NanoShaper Patcher [2] to patch DelPhi.

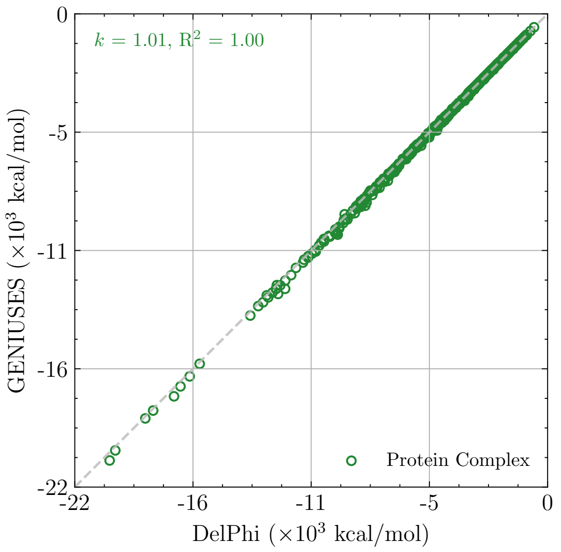

Figure S8: Energy comparison with DelPhi.

Regarding the energy comparison, we compared the reaction energies of the protein complex dataset provided by GENIUSES with those computed using DelPhi. As shown in Figure S8, the atomic energies reach a minimum of only -22,000 kcal/mol. This limitation is attributed to DelPhi encountering a "core dump" issue when calculating larger molecules within the protein complex dataset. Consequently, results are available for only 492 out of 622 molecules in the dataset. However, as indicated in Figure S8, the energy values obtained from the GENIUSES model exhibit a perfect agreement with those from DelPhi, demonstrating a high correlation with $R^2 = 1$.

References

(1) Case, D.; Aktulga, H.; Belfon, K.; Ben-Shalom, I.; Berryman, J.; Brozell, S.; Cerutti, D.; Cheatham, T.; Cisneros, G.; Cruzeiro, V.; others Amber 2022, University of California:

[2] `https://electrostaticszone.eu/downloads/scripts-and-utilities/22-delphi-patcher.html`

San Francisco, CA, USA. 2022.

(2) Nagelkerke, N. J.; others A note on a general definition of the coefficient of determination. *biometrika* **1991**, *78*, 691–692.

(3) Willmott, C. J.; Matsuura, K. Advantages of the mean absolute error (MAE) over the root mean square error (RMSE) in assessing average model performance. *Climate research* **2005**, *30*, 79–82.

(4) Paszke, A.; Gross, S.; Massa, F.; Lerer, A.; Bradbury, J.; Chanan, G.; Killeen, T.; Lin, Z.; Gimelshein, N.; Antiga, L.; others Pytorch: An imperative style, high-performance deep learning library. *Advances in neural information processing systems* **2019**, *32*.

(5) Connolly, M. L. Analytical molecular surface calculation. *Journal of applied crystallography* **1983**, *16*, 548–558.

(6) Connolly, M. L. Solvent-accessible surfaces of proteins and nucleic acids. *Science* **1983**, *221*, 709–713.

(7) Lee, B.; Richards, F. The Interpretation of Protein Structures: Estimation of Static Accessibility. *55*, 379–IN4.

(8) Xu, D.; Zhang, Y. Generating triangulated macromolecular surfaces by Euclidean distance transform. *PloS one* **2009**, *4*, e8140.

(9) Decherchi, S.; Rocchia, W. A general and robust ray-casting-based algorithm for triangulating surfaces at the nanoscale. *PloS one* **2013**, *8*, e59744.

(10) Wang, J.; Cai, Q.; Xiang, Y.; Luo, R. Reducing grid dependence in finite-difference Poisson–Boltzmann calculations. *Journal of chemical theory and computation* **2012**, *8*, 2741–2751.

(11) You, T.; Bashford, D. An analytical algorithm for the rapid determination of the solvent accessibility of points in a three-dimensional lattice around a solute molecule. *Journal of Computational Chemistry* **1995**, *16*, 743–757.

(12) Rocchia, W.; Sridharan, S.; Nicholls, A.; Alexov, E.; Chiabrera, A.; Honig, B. Rapid grid-based construction of the molecular surface and the use of induced surface charge to calculate reaction field energies: Applications to the molecular systems and geometric objects. *Journal of computational chemistry* **2002**, *23*, 128–137.

(13) Wei, H.; Zhao, Z.; Luo, R. Machine-Learned Molecular Surface and Its Application to Implicit Solvent Simulations. *Journal of chemical theory and computation* **2021**, *17*, 6214–6224.

(14) Case, D. A.; Skrynnikov, N. R.; Cheatham III, T. E.; Mikhailovskii, O.; Simmerling, C.; Xue, Y.; Roitberg, A.; Xue, Y.; Roitberg, A.; Izmailov, S. A.; Merz, K. M.; Kasavajhala, K.; others *AMBER 23 Reference Manual*; University of California, 2023.
