# Supplementary material for: Grid-Robust Efficient Neural Interface Model for Universal Molecule Surface Construction from Point Clouds
Source: J Phys Chem Lett. 2023 Oct 2;14(40):9034–41. doi: 10.1021/acs.jpclett.3c02176 (PMC10577766; doi:10.1021/acs.jpclett.3c02176)
Supplement: Supplementary file 2 — jz3c02176_si_002.pdf [file jz3c02176_si_002.pdf]

jz-2023-021769.R1

Name: Peer Review Information for "Grid-robust Efficient Neural Interface Model for Universal Molecule Surface Construction from Point Clouds"

First Round of Reviewer Comments

Reviewer: 1

Comments to the Author

This seems like a clever and useful application of AI to a common and significant challenge in physical modeling of molecules. My only recommendations have to do with clarity of presentation.

This reader at least would be helped by addition of a brief paragraph explaining the basic idea of the method at a high level.

1) Figure 1a: this looks like one dark helix surrounded by light helices, but I guess it is supposed to represent a molecular structure whose surface is shown in 1d. It would be good to clarity this figure. Also, even if GENIUSES does well with coarse grids, the grid in Figure 1a looks much to coarse to give a useful surface, given that 5 turns of an alpha helix fit into one grid box.

Page 5 line 3, and elswhere: this mentions Ng grids. but a grid normally is a lattice with many edges and vertices. I guess instead of "grids" the authors mean "grid points"--? Would be good to clarify this.

Eq 1: I'm not clear what R^+ and R^- represent. I should probably understand this notation, but maybe other readers also could use help with it, so I recommend explaining it.

Page 7, line 29: it would be helpful to state here what the "ground truth" result will be.

Reviewer: 2

Comments to the Author

The manuscript addresses an important problem, namely the construction of molecular surface of biological macromolecules and thus it is an timely contribution to the field of computational chemistry. The manuscript demonstrates that the method is robust and very efficient, achieving a speed up of 15 compared to classical molecular surface builder algorithms. The method is implemented in AMBER and thus it is accessible to the community. I have only several minor comments which I will leave up to the editor and the authors.

Major comments: None

Minor comments:

1) In the intro, the authors may want to mention quire efficient method for constructing molecular surface

https://pubmed.ncbi.nlm.nih.gov/11913378/

2) Perhaps one want to clarify that not all PB methods require molecular surface. Recently a surface-free PB was reported and treats the solute and solvent on the same footage:

https://pubmed.ncbi.nlm.nih.gov/23585741/

Reviewer: 3

Comments to the Author

The present work proposes a general framework to construct solvent exluded surface (SES) based on point clouds and nueral networks. The methodology appears to offer significant computational speedups without losing accuracy. The results suggest that the model can be robust against grid size which translates to scalability. This framework model will be a new and significant contribution to the field of computational chemistry and biology. Therefore, I can recommend the manuscript for publication in the Journal of Physical Chemistry Letters as is.

Reviewer: 4

Comments to the Author

The Authors present a ML-based approach to approximate the MS of a biomolecular system. One of the biggest advantages I see is the leveraging of library availability, which allows several implementations, namely Fortran, Torch CPU, Torch CUDA, and CUDA to meet different utilization scenarios.

In contrast, I do not think it is proved that the approach presented in the manuscript is cutting edge.

In the manuscript it is mentioned more than 16 times, as a reference, the "classical SES". But there is no "classical SES", the SES is one possible definition of the MS, and this definition dates back to the 70-ies (Lee and Richards). The first implementation, to my knowledge, was from Connolly and one of the most widely adopted has been for years the one from Sanner (MSMS). So the Authors maybe want to say "classical implementations of the SES", which would be more appropriate. More recently, efficient and robust implementations of the "classical" SES, to my knowledge, are EDTsurf and NanoShaper, the latter has been applied also to quite large systems.

When I go to the SI to see what the Authors mean as the "classical SES", I only find the MLSES approach, which is, similarly to theirs, ML based.

I think that in order to prove that their approach is outstanding among the others, they must also compare against some really "classical implementation", meaning not ML based.

In addition, several aspects, that would really be of interest, are overlooked. For instance, the locality of the approach, I guess this approach would be particularly suitable to adapt a previously built MS to a new one deriving from a change of a rotamer in a residue. Most existing methods are "global" in the sense that they would need to rebuild everything even for a local modification. The Author say that a neighbourhood of 24 atoms is sufficient to perform the local prediction, so I think their approach is promising in this sense.

Moreover, some PB solvers, such as DelPhi, get a relatively good robustness concerning the reaction field energy by projecting points onto a semi-analytical description of the MS. How would this be possible with the present approach?

The surface representation part seems to be unnecessarily complex. It should be made simpler and clearer. For instance when they say:

"The concept of point cloud was introduced here to improve the representation of 3D

molecular coordinates, where it consists of a set of data points with each point denoting

the specific position of atoms in molecular systems"... does this mean that the A quantity defined in Eq. 2 is simply the set of atom centers? I think the points are located in atom centers? Or are them on the atomic surface? a sampling of their volume? please clarify.

The comparison of SES as done in the point clouds of figure 3 is too qualitative. The SES has several peculiarities, such as self-intersections, that should be discussed and that cannot be assessed by visual inspection.

What does the approach do with the interior regions? Are there cavities? The ML algorithm could be trained also on them, starting from a "classical implementation". In principle I don't see any difference, so why the approach is weaker in these cases?

What is the PB solver used in pairing with GENIUSES?

In the robustness section, the Authors seem to be surprised that a model trained on proteins performs well also on nucleic acids and protein complexes, why? The construction of the SES is agnostic with respect to chemistry, it starts from a union of partially overlapping balls. So, since the radii of the atoms are similar and also the bond lengths are similar, also the surface should be predicted in a similar way, or not?

Minor things:

This sentence seems inconsistent:

"For systems containing fewer than 2000

atoms, regardless of the platform or library utilized, our method significantly outperforms

the classical SES and MLSES. However, when the atom count exceeds 2000, only the method

implemented with Kernel Fortran on a CPU platform exhibits a marginally slower speed than

MLSES implemented on GPU."

I would not say "grid size of 0.35A" but "grid spacing of 0.35A"

Author's Response to Peer Review Comments:

We extend our sincere gratitude for your prompt response and the kind consideration of our manuscript. We appreciate the valuable comments and suggestions from four reviewers on revision of the

manuscript. We have carefully addressed all comments and critiques accordingly. Specifically, we have added timing comparisons with other classical implementations of solvent-excluded surface methods, namely NanoShaper and EDTSurf. Additionally, we have updated all figures and expanded discussions in both the manuscript and the Supporting Information.

We have undertaken a comprehensive revision of our manuscript, marking the modifications in blue. We have also ensured the text is grammatically accurate and have clarified any ambiguous abbreviations. All non-scientific suggestions have been thoughtfully integrated.

We sincerely thank the editor and all reviewers for their valuable comments and suggestions that have been incorporated to improve the quality of our revised manuscript. In the following, the reviewer comments are shown in italic. Our responses are shown in blue.

For Reviewer #1

This seems like a clever and useful application of AI to a common and significant challenge in physical modeling of molecules. My only recommendations have to do with clarity of presentation. This reader at least would be helped by addition of a brief paragraph explaining the basic idea of the method at a high level.

Comments from review #1:

Figure 1a: this looks like one dark helix surrounded by light helices, but I guess it is supposed to represent a molecular structure whose surface is shown in 1d. It would be good to clarity this figure. Also, even if GENIUSES does well with coarse grids, the grid in Figure 1a looks much to coarse to give a useful surface, given that 5 turns of an alpha helix fit into one grid box.

Response:

Thank you for your suggestion. More statements were added in the revised manuscript and Figure 1 was redrawn for clarity. In this figure, we only want to highlight the workflow for predicting surface given a specific molecular structure. In Figure 1a, the grids points and spacing (s) shown here are just for illustration. Besides, for better depicting the preprocessing step (Figure 1b), only one helix is shown in detail while the protein is shown in transparent cartoon. In realistic applications, the grid spacing (s) is much smaller than the one shown here and the values tested in this work are all less than 1 Å (at atomic level).

Revision made in text: (Page 4)

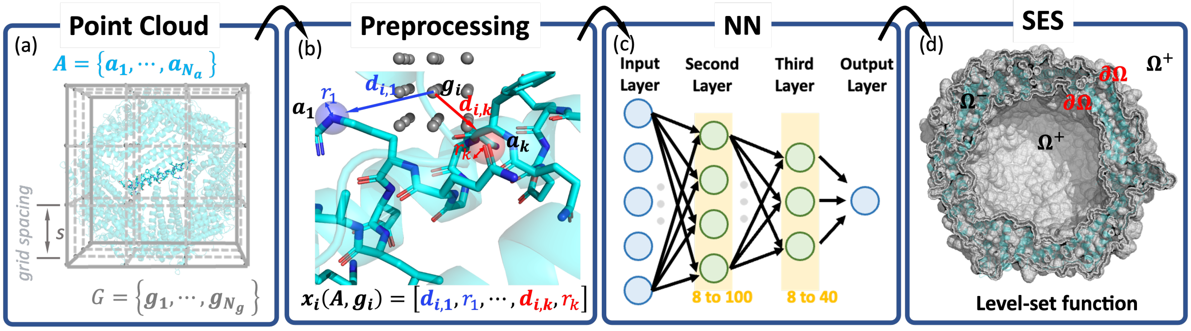

Figure 1: Schematic illustration of the workflow: (a) point cloud setup, digital representation of a specific molecule; (b) pre-processing the point cloud to fulfill the invariance of surface with respect to the translation and rotation of the molecule and insensitivity to the pre-defined grid spacing; (c) construction of neural network (NN) for fitting the level-set function; (d) construction of SES surface using the level-set function. Here, the point cloud presentation of a helical protein is illustrated with an artificially coarse grid. One specific helix of the protein is highlighted for subsequent processing.

Comments from review #1:

Page 5 line 3, and elsewhere: this mentions Ng grids. but a grid normally is a lattice

with many edges and vertices. I guess instead of "grids" the authors mean "grid points"? Would be good to clarify this.

Response:

Thank you for pointing out the ambiguity. Here "Ng grids" actually means "grid points" of the lattice. We have revised the terminology throughout the manuscript to ensure consistency and clarity, replacing "grids" with "grid points" as appropriate. We appreciate your attention to detail.

Revision made in text: (Page 4)

Within this framework, the entire surface is discretized into a three-dimensional (3D) grid space $\boldsymbol{G} = \{\mathbf{g}_1, \ldots, \mathbf{g}_{N_g}\}$ containing N_g grid points, where $\mathbf{g}_i \in \mathbb{R}^3$ signifies the coordinate of the i-th point in the 3D space. The distance between two nearest neighbor points is a constant value $s \in (0, 1]$ (also referred to as the grid spacing, Figure 1(a)). The sign of level-set values (y_i) indicates whether a grid point $\mathbf{g}_i$ is positioned outside-of-boundary or inside-of-boundary [1].

Revision made in text: (Page 12)

$\mathrm{N_g}$ is the number of total grid points for a given target molecule under the current grid spacing.

Comments from review #1:

Eq 1: I'm not clear what R^+ and R^- represent. I should probably understand this notation, but maybe other readers also could use help with it, so I recommend explaining it.

Response:

Thank you for raising the concern regarding the notation in Eq 1. R^+ and R^- are indeed specific to our formulation, and we apologize for the oversight in not explaining them explicitly. We have added a detailed explanation to explain the meaning and significance of both $\mathbb{R}^+$ and $\mathbb{R}^-$. We hope this clarification will make the equation more accessible to all readers.

Revision made in text: (Page 4-5)

The sign of level-set values (y_i) indicates whether a grid point $\mathbf{g}_i$ is positioned outside-of-boundary or inside-of-boundary [1] (as illustrated in Figure 1(d)),

$$y_i = \begin{cases} \mathbb{R}^+, & \mathbf{g}_i \in \text{exterior region } \Omega^+ \\ 0 \;\;, & \mathbf{g}_i \in \text{on surface } \partial\Omega \\ \mathbb{R}^-, & \mathbf{g}_i \in \text{interior region } \Omega^- \end{cases} \tag{1}$$

where Ω^+ represents the exterior surface region and Ω^- corresponds to the interior region, $\mathbb{R}^+$ and $\mathbb{R}^-$ are corresponding positive and negative level-set values, respectively. The sign transition between the inside and outside is made at the point $\partial\Omega$, referred to as the surface. The surface, therefore, can be ascertained by identifying the phase of sign alteration.

Comments from review #1:

Page 7, line 29 it would be helpful to state here what the "ground truth" result will be.

Response:

Thank you for the valuable suggestion. We have added a statement to explain the "ground truth" result. This inclusion will indeed make the subsequent discussion and analysis more coherent and easier to follow for readers.

Revision made in text: (Page 7)

Utilizing the aforementioned input features ($\mathbf{x}_i$) and objective (Figure 1(d)), the task can be generalized as below,

$$\hat{y}_i = \hat{\phi}\left(\mathbf{x}_i(\boldsymbol{A}, \mathbf{g}_i); \theta\right). \tag{2}$$

where θ symbolizes the trainable parameters of the neural network. The aim of the training objective function is to minimize the disparity between the ground-truth level-set value y_i that directly extracted from the AMBER PBSA benchmark suite and the prediction $\hat{y}_i$

=========The end of reply to reviewer #1 ===========

For Reviewer #2

The manuscript addresses an important problem, namely the construction of molecular surface of biological macromolecules and thus it is an timely contribution to the field of computational chemistry. The manuscript demonstrates that the method is robust and very efficient, achieving a speed up of 15 compared to classical molecular surface builder algorithms. The method is implemented in AMBER and thus it is accessible to the community. I have only several minor comments which I will leave up to the editor and the authors.

Comments from review #2:

In the intro, the authors may want to mention a quite efficient method for constructing molecular surface *https://pubmed.ncbi.nlm.nih.gov/11913378/*

Response:

Thank you for highlighting this important reference. We acknowledge the significance and efficiency of the method presented in the mentioned paper for constructing molecular surfaces. We have mentioned the method in the introduction and provided appropriate citations. This will not only enrich our discussion but also offer the readers a broader perspective on the topic.

Revision made in text: (Page 2)

For example, a refined density function strategy founded on a modified vdW surface was suggested for numerical Poisson-Boltzmann applications [2]. Taking both accuracy and efficiency into consideration, an analytic surface representation was generated in advance and then mapped onto arbitrary lattices [3–5]. Such strategy and algorithm were further optimized and streamlined by Rocchia et al. [6] Concurrently, the field-view method was utilized for SES or SAS generation under the finite-difference scheme [7].

Comments from review #2:

Perhaps one want to clarify that not all PB methods require molecular surface. Recently a surface-free PB was reported and treats the solute and solvent on the same footage: *https://pubmed.ncbi.nlm.nih.gov/23585741/*

Response:

Thank you for bringing to our attention the recent developments in surface-free PB methods. We agree that it is essential to give readers a comprehensive view of the field. We have clarified in the manuscript that while many PB methods utilize molecular surfaces, there are notable exceptions, like the surface-free PB method cited. We have referenced the provided paper and discuss the unique approach of treating the solute and solvent on equal footing. This will ensure that our readers are informed of the various methodologies available in the field.

Revision made in text: (Page 3))

It is worth noting that a surface-free Poisson-Boltzmann solver model treats the solute and solvent uniformly, bypassing the necessity of generating a molecular surface [8].

=========The end of reply to reviewer #2 ===========

For Reviewer #3

The present work proposes a general framework to construct solvent exluded surface (SES) based on point clouds and nueral networks. The methodology appears to offer significant computational speedups without losing accuracy. The results suggest that the model can be robust against grid size which translates to scalability. This framework model will be a new and significant contribution to the field of computational chemistry and biology. Therefore, I can recommend the manuscript for publication in the Journal of Physical Chemistry Letters as is.

Response:

Thank you for your thorough review and positive feedback on our work. We appreciate your recognition of the potential impact of our method in the fields of computational chemistry and biology. We will be active on this project to further improve its efficiency, scalability, and accuracy.

==========The end of reply to reviewer #3 ============

For Reviewer #4

The Authors present a ML-based approach to approximate the MS of a biomolecular system. One of the biggest advantages I see is the leveraging of library availability, which allows several implementations, namely Fortran, Torch CPU, Torch CUDA, and CUDA to meet different utilization scenarios. In contrast, I do not think it is proved that the approach presented in the manuscript is cutting edge.

Comments from review #4:

In the manuscript it is mentioned more than 16 times, as a reference, the "classical SES". But there is no "classical SES", the SES is one possible definition of the MS, and this definition dates back to the 70-ies (Lee and Richards). The first implementation, to my knowledge, was from Connolly and one of the most widely adopted has been for years the one from Sanner (MSMS). So the Authors maybe want to say "classical implementations of the SES", which would be more appropriate. More recently, efficient and robust implementations of the "classical" SES, to my knowledge, are EDTsurf and NanoShaper, the latter has been applied also to quite large systems.

Response:

Thank you for the detailed feedback and historical perspective on the Solvent Excluded Surface (SES) definition and its various implementations. We understand and acknowledge the oversight in our usage of the term "classical SES."

We agree that our reference to "classical SES" should more accurately be "classical implementations of the SES." We have made this correction throughout the manuscript. In this work, we employed the classical implementation method of AMBER/PBSA for the purpose of training and benchmark,[9] following the basic ideas of Connolly's surface definition[10] and Rocchia's approach[6]. To be specific, some statements were added in the revised manuscript.

Revision made in text: (Page 1-2)

Compared to the classical implementation of SES in the AMBER software package, our framework achieved a 26-fold speedup while retaining ~ 95 % accuracy when ported to the GPU platform using CUDA.

Revision made in text: (Page 3-4)

In this study, we utilize three distinct datasets. For model training, we employ a set of 573 proteins derived from the AMBER PBSA benchmark suite. This dataset comprises biomolecules with atomic counts ranging from 377 to 8254, offering a diverse array of geometries. The training and benchmarked data for our model was derived from the AMBER/PBSA surface builder, which is tailored for the geometry-based SES, herein denoted as "classical SES".[9] This approach follows the fundamental principles articulated by You [5] and Rochia et al.[6].

Revision made in text: (Page 13-14)

Using the build-in classical SES procedure in AMBER/PBSA as a benchmark, we also compared the time consumption among widely used classical implementations of SES methods, namely EDTSurf [11] and NanoShaper [12], as well as the machine-learned method MLSES with our model implemented across various platforms and libraries. For a fair comparison, we disabled the printing of intermediate grid points information in both AMBER/PBSA and GENIUSES, only retaining the printing of surface information, consistent with other SES programs. As shown in Figure 5(a), with increasing the number

of atoms, all methods tested in this work show a consistent trend. This trend coincides with our intuition that the task of surface construction is proportional to the number of atoms. For systems containing fewer than 2000 atoms, regardless of the platform or library utilized, our method (solid circle) significantly outperforms the classical SES (blue "X") and MLSES (pink "X"). When the atom count exceeds 2000, most implementations of the method still outperforms the classical SES (blue "X") and MLSES (pink "X"), except that implemented with the Kernel Fortran on CPU platform (green solid circle), which exhibits a marginally slower speed than MLSES (pink "X").

Comments from review #4:

When I go to the SI to see what the Authors mean as the "classical SES", I only find the MLSES approach, which is, similarly to theirs, ML based.

I think that in order to prove that their approach is outstanding among the others, they must also compare against some really "classical implementation", meaning not ML based.

Response:

Thank you for your insightful comments, which have greatly helped improve our manuscript. In response to your concerns regarding the comparison of our approach to the "classical implementation of SES," we have addressed them as follows:

1. **Clarification on Classical SES:** To ensure clarity, we wish to clarify that our method has indeed been compared with the Classical implementation of SES in AMBER/PBSA.[9] This implementation, as you correctly pointed out, is not ML-based and follows the basic ideas as outlined by You[5] and Rocchia et al.[6]. This comparison with a non-ML based implementation aims to provide a robust assessment of our technique vis-à-vis classical methods.

2. **Addition of Benchmark Results with EDTsurf and NanoShaper:** We concur with your suggestion that benchmarking against more classical, non-ML based methods would enhance the comprehensiveness of our study. We have added additional results comparing our method against EDTsurf [11] and NanoShaper [12], two advanced MS solutions for large-scale systems. We have added the detailed experiment data and discussion in the revised main text and SI section S6.2 Inference Time.

We hope these additions and clarifications will resolve the concerns you raised. We are deeply grateful for your thorough review and constructive feedback, which have been instrumental in revising the manuscript.

Revision made in text: (Page 13-15)

Using the build-in classical SES procedure in AMBER/PBSA as a benchmark, we also compared the time consumption among widely used classical implementations of SES methods, namely EDTSurf [11] and NanoShaper [12], as well as the machine-learned method MLSES with our model implemented across various platforms and libraries. For a fair comparison, we disabled the printing of intermediate grid points information in both AMBER/PBSA and GENIUSES, only retaining the printing of surface information, consistent with other SES programs. As shown in Figure 5(a), with increasing the number of atoms, all methods tested in this work show a consistent trend. This trend coincides with our intuition that the task of surface construction is proportional to the number of atoms. For systems containing fewer than 2000 atoms, regardless of the platform or

library utilized, our method (solid circle) significantly outperforms the classical SES (blue "X") and MLSES (pink "X"). When the atom count exceeds 2000, most implementations of the method still outperforms the classical SES (blue "X") and MLSES (pink "X"), except that implemented with the Kernel Fortran on CPU platform (green solid circle), which exhibits a marginally slower speed than MLSES (pink "X").

The efficiency of our method is further quantified by the relative speed compared to the classical SES@AMBER, as shown in Figure 4(b) and Table 1. The relative speedup average is calculated by taking the average of relative speedup on the each molecule between the different SES programs and the benchmark. From this, we can safely conclude that 26-fold speedup of our model with respect to the classical SES@AMBER could be achieved over protein dataset when utilizing Kernel CUDA. (Table 1) Even with a CPU implementation (LibTorch GENIUSES), our model still exhibits a five-fold speedup compared to the classical SES. These results were consistently observed over two other datasets, one of which includes much larger protein complex structures (Figure S7). Comparison was further conducted over widely used classical implementations of SES methods, specifically EDTSurf and NanoShaper. As detailed in Table 1, the EDTSurf method exhibits a slightly slower surface construction rate compared to the benchmark method with a speedup of 0.37 and 0.66 over nucleic acid and protein dataset, respectively. For NanoShaper, an eight-fold speedup was achieved over protein dataset when utilizing only one thread. Such speedup increases to around 19 when applying 32 threads. Further increasing threads up to 64 does not significantly accelerate its speed. The surface generation speed is highly dependent on the molecular size.[12] A comprehensive comparison over large-scale protein complexes was conducted and listed in Table 1. Both EDTSurf and NanoShaper outperform the classical SES implemented in AMBER, the speedup for EDTSurf is 2.15 and 18.16 for NanoShaper when utilizing 64 threads. For our method, its scalability was further demonstrated by a remarkable relative speedup of 33.28 over dataset protein complex when applying CUDA platform. Given these findings, we further envision that the method could be used in the process of drug screening where computational speed is a critical factor. In a concerted effort to benefit the broader research community, the efficient implementation of our proposed method has been integrated into the widely-used molecular modeling software package, AMBER [13].

Table 1: Comparative analysis of performance with average run time and relative speedup for the nucleic acids, protein, and protein complex dataset among different SES programs. All speedup are with respect to the Classical SES from AMBER/PBSA.

Methods	CPU	GPU	Avg. Time (Std.) (s)	Rel. Speedup	Avg. Time (Std.) (s)	Rel. Speedup	Avg. Time (Std.) (s)	Rel. Speedup
			nucleic acid		protein		protein complex	
Classical SES@AMBER	1	0	4.45 (3.32)	1.00	8.30 (4.77)	1.00	35.07 (42.94)	1.00
GENIUSES Torch@CPU	1	0	0.88 (0.62)	5.02	1.54 (0.92)	5.44	6.87 (8.09)	4.87
GENIUSES Torch@GPU	1	1	1.16 (0.40)	3.61	1.03 (0.21)	7.64	1.67 (1.17)	17.52
GENIUSES CUDA@GPU	1	1	0.19 (0.15)	23.38	0.33 (0.20)	25.55	1.00 (1.18)	33.28
EDTSurf	1	0	14.69 (4.68)	0.37	14.09 (4.42)	0.66	15.32 (6.59)	2.15
NanoShaper 1 Thread	1	0	0.68 (0.45)	6.42	1.02 (0.50)	7.97	5.27 (8.35)	7.74
NanoShaper 32 Threads	32	0	0.27 (0.21)	16.98	0.43 (0.25)	19.30	2.43 (3.65)	15.86
NanoShaper 64 Threads	64	0	0.27 (0.21)	16.81	0.44 (0.25)	19.22	2.05 (2.89)	18.16

Revision made in text: (SI Page S8)

Among these, the classical implementation of solvent-excluded surface (SES) approaches in AMBER/PBSA [10, 14, 15], EDTSurf [11] and NanoShaper [12] stand out as well-researched and widely used strateges in computational chemistry and molecular modeling for representing a molecule's surface in relation to its interaction with a solvent. The build-in classical implementation of SES method in AMBER/PBSA is specifically designed for geometry-based SES the geometry-based SES, referred to as "classical SES" [9]

in this paper, following the basic ideas of Connolly's surface definition[10] and the foundational principles outlined by You[5] and Rocchia et al.[6]. The SES is essentially a smooth, continuous boundary that encapsulates the volume inaccessible to solvent molecules. In the classical implementation of SES methods, ϕ is determined by rolling a probe sphere, simulating the solvent molecule, over the van der Waals surface of the solute molecule. The radius of this probe sphere is typically approximated to the size of a water molecule, around 1.4 Å.

Despite its ability to provide accurate surface estimations, the classical implementation of SES methods can be computationally demanding and time-intensive, especially for large systems or when high-resolution surface representations are required.

Comments from review #4:

In addition, several aspects, that would really be of interest, are overlooked. For instance, the locality of the approach, I guess this approach would be particularly suitable to adapt a previously built MS to a new one deriving from a change of a rotamer in a residue. Most existing methods are "global" in the sense that they would need to rebuild everything even for a local modification. The Author say that a neighbourhood of 24 atoms is sufficient to perform the local prediction, so I think their approach is promising in this sense.

Response:

Thank you for highlighting the potential advantage of our method concerning its locality. We concur with your assessment. Given the localized nature of our approach, it is indeed more suitable for adapting a previously built molecular surface (MS) to changes resulting from local modifications, such as rotamer shifts in residues. This is in contrast to many existing "global" methods that necessitate a complete rebuild even for minor alterations.

We have emphasized this aspect further in our manuscript and provided a dedicated discussion on the advantages of our method in scenarios involving local modifications to the molecular structure. We believe this insight is crucial and will add significant value to our paper. Your constructive feedback is much appreciated.

Revision made in text: (Page 6)

In order to achieve surface invariance, the relative distance was adopted as the feature of our model. Moreover, the coordinates of the nearest-k atoms $\{\mathbf{a}_1, \ldots, \mathbf{a}_k\}$ around the queried grid $\mathbf{g}_i$, along with their corresponding radii, were chosen as the surrounding environments. This was done to improve the representation and decrease the grid-spcaing dependency. Additionally, this makes our method more suitable for adapting changes from local modifications, such as rotamer shifts in residues, given a previously built surface. Such features could further shorten the time consumption in the surface generation and are distinct from present other methods that necessitate a complete rebuild.

Comments from review #4:

Moreover, some PB solvers, such as DelPhi, get a relatively good robustness concerning the reaction field energy by projecting points onto a semi-analytical description of the MS. How would this be possible with the present approach?

Response:

Thank you for raising an insightful question concerning the projection of points onto a semi-analytical description of the MS, especially in the context of PB solvers like DelPhi.

It is widely noted that some PB solvers demonstrate commendable robustness in the context of reaction field energy by leveraging such projections. Both AMBER/PBSA and DelPhi have the capability to efficiently project the polarization charges onto the SES. This is achieved by maintaining a vast amount of information during the construction of the classical SES. In particular, by keeping track of the solvent probe or the solute atom sphere accountable for a given polarization charge, we can seamlessly project this polarization charge onto the SES, as described in our previous work on the classical SES implementation[9]

However, the method's integrity could be a subject of debate. Specifically, the assignment of the remembered solvent probe or solute atom sphere – or what we might term its "owner" – isn't always unequivocally correct. In the classical approach to computing the SES, challenges frequently emerge because analytical methods are utilized based on predefined rules rather than producing a specific differentiable function. This often results in situations where the gradient direction cannot be determined. Such uncertainties can introduce complexities into numerical PB calculations.

Therefore, one of the advantages of using machine learning (ML) to construct the Solvent Excluded Surface (SES) is that our model is fully differentiable. With known weights and biases, we can perform differentiation with respect to the input coordinates (x, y, z) and calculate the gradient of these input features. Consequently, we can extend the GENIUSES model to compute the second derivative with respect to x, y, and z, enabling us to calculate the surface curvature.

The suggestion highlights a subtle aspect in applying our method, and we value this chance to clarify. Nevertheless, it should also be pointed out that our level-set-based model is differentiable and can in principle be extended to realize the same strategy that is currently feasible in the classical SES method.

Regarding the energy comparison, we compared the reaction energies of the protein complex dataset provided by GENIUSES with those computed using DelPhi. As shown in Figure 2, the atomic energies reach a minimum of only -22,000 kcal/mol. This limitation is attributed to DelPhi encountering a "core dump" issue when calculating larger molecules within the protein complex dataset. Consequently, results are available for only 492 out of 622 molecules in the dataset. However, as indicated in Figure 2, the energy values obtained from the GENIUSES model exhibit a near perfect agreement with those from DelPhi, demonstrating a high correlation with $R^2 = 1$. We have also added this results to the SI S6.3.

Revision made in SI: (Page S13-S14)

To further understand the benefit of our GENIUSES method to the AMBER PBSA, we conduct a timing experiment on comparing the inference performance of our AMBER PB solver (PBSA) against another state-of-the-art PB solver, DelPhi, where NanoShaper is proposed to speed up the DelPhi program by accelerating the molecule surface building process. For the NanoShaper, we compile the latest release version 0.7 and follow the instruction provided in NanoShaper Patcher to patch DelPhi.

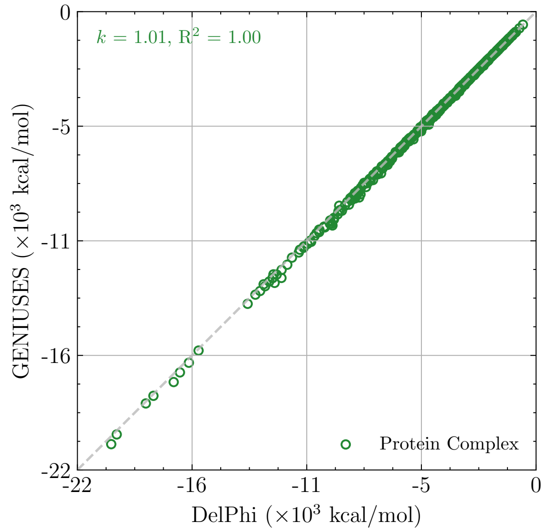

Figure 2: Energy comparison with DelPhi.

Comments from review #4:

The surface representation part seems to be unnecessarily complex. It should be made simpler and clearer. For instance when they say: "The concept of point cloud was introduced here to improve the representation of 3D molecular coordinates, where it consists of a set of data points with each point denoting the specific position of atoms in molecular systems"... does this mean that the A quantity defined in Eq. 2 is simply the set of atom centers? I think the points are located in atom centers? Or are them on the atomic surface? a sampling of their volume? please clarify.

Response:

Thank you for highlighting the ambiguity in our description of the surface representation. We apologize for the confusion and understand the need for clarity in this section. We have further improved our writings to make it straight forward and more intuitive.

To clarify: The "point cloud" concept is indeed utilized to enhance the representation of 3D molecular coordinates. In our context, each point in the point cloud corresponds to the center of an atom within the molecular system. Thus, the quantity A defined in Eq. 2 represents the set of atom centers.

However, we understand that our initial wording could be interpreted in multiple ways. We have restructured this section to ensure a more straightforward and precise explanation. Specifically, we have explicitly stated the relationship between the point cloud points and the atom centers, removing any ambiguity.

Revision made in text: (Page 5)

The concept of point cloud was introduced here for the representation of 3D molecular coordinates,[16–18] where it consists of a set of data points with each point denoting the specific position of atoms in molecular systems. For a molecule containing N_a atoms, it can be expressed in digital format ($\mathbf{A}$)

$$\boldsymbol{A} = \{\mathbf{a}_1, \ldots, \mathbf{a}_{N_a}\} \tag{3}$$

where $\mathbf{a}_i \in \mathbb{R}^3$ represents point located in the center of atom i within a specified 3D space.

Comments from review #4:

The comparison of SES as done in the point clouds of figure 3 is too qualitative. The SES has several peculiarities, such as self-intersections, that should be discussed and that cannot be assessed by visual inspection.

Response:

Thank you for highlighting the importance of providing a more quantitative evaluation of the SES beyond the visual representations. Throughout the main text, we employed two evaluation method that widely used in 3D computer graphic (**CD** score and **F** score) to quantify the performance of our model. To improve the readability, Figure 3 was reorganized and some discussions on the self-intersections were included in our main text.

Revision made in text: (Page 10)

Upon investigating less accurate cases, we identified that such errors mainly come from the interior region where self-intersection could occur between internal cavity and an accessible region.[12] (Figure S4) Such inaccuracy mainly comes from the imbalance between data distribution of the exterior and interior. (Figure S1)

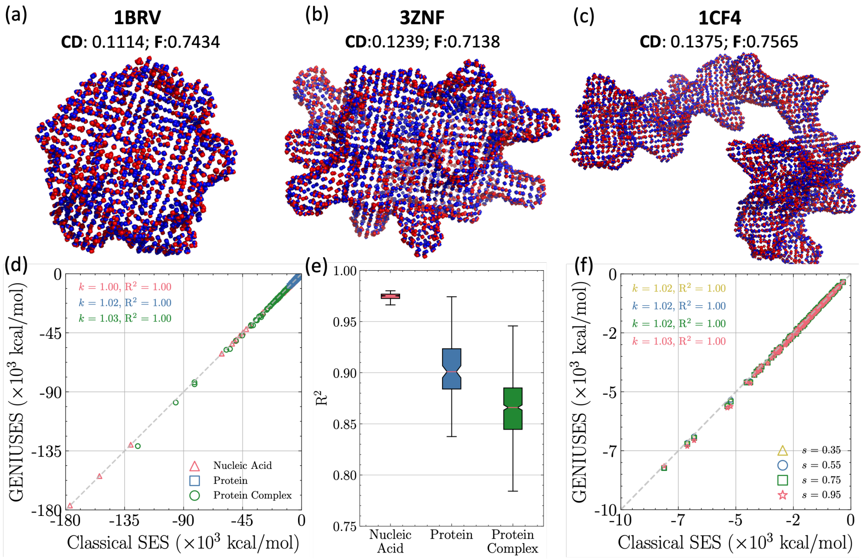

Figure 3: Model accuracy and robustness with different test systems and grid spacings. (a-c) Superimposed surface generated by GENIUSES (blue) and classical SES (red) for representative molecular structures, corresponding PDB ID's and metric values are also shown; (d) Comparison of PB reaction field energies across three different datasets with classical SES surface and GENIUSES surface. Here grid spacing is set to be 0.35 Å; (e) R-square values of GENIUSES for the three datasets; (f) Comparison of PB reaction field energies between the surface predicted by GENIUSES with different grid spacings ($s = 0.35, 0.55, 0.75, 0.95$ Å) and classical SES surface with 0.35 Å grid spacing.

Comments from review #4:

What does the approach do with the interior regions? Are there cavities? The ML algorithm could be trained also on them, starting from a "classical implementation". In principle I don't see any difference, so why the approach is weaker in these cases?

Response:

Thank you for bringing up the valuable query about our method's treatment of interior regions, specifically concerning cavities.

Our approach does identify cavities and isolated surfaces within the interior regions. Upon closely examining the prediction visualization, we have noticed that our model tends to predict channels that seem to connect the outer surface to these stand-alone island surfaces within the cavities. Such predictions hint towards the model's attempt to reconcile with the sparse data representations of these intricate internal structures.

In terms of data distribution, the interior regions are significantly underrepresented in comparison to the exterior regions, making up only about 3 % of the dataset. Given the data-driven nature of machine learning, this imbalance poses a challenge. When trained on such skewed datasets, machine learning models might find it arduous to robustly understand and predict behaviors associated with the less represented classes or features.

Your suggestion of incorporating a "classical implementation" for the training of the ML algorithm, especially for these interior regions, is well taken. Indeed, the fundamentals of machine learning would not differentiate between interior and exterior regions. It's primarily the dataset distribution that causes the differential performance. Enriching our ML training with patterns and insights from classical methods might be a promising avenue to enhance its performance for the interior regions, and we are grateful for this suggestion.

To provide further clarity on this matter, we have detailed the distribution of the interior grid points in our Supplementary Information under the S1.3 "Interior Points Distribution" section.

In essence, the sparse representation of the interior regions in our dataset is the primary reason for our model's seemingly weaker performance in these areas. However, your feedback has illuminated potential directions to strengthen our approach, and we are earnestly considering them for our next steps.

Revision made in supporting information: (SI Page S4)

S1.3 Interior Points Distribution

We investigate the distribution of interior grid points within the complete set of grid samples from the protein dataset. As depicted in Figure 4, the average proportion of interior points is consistently below 3.10% throughout the dataset.

Revision made in text: (Page 10)

Besides, its classification task introduces sensitivity in surface construction. Upon investigating less accurate cases, we identified that such errors mainly come from the interior region where self-intersection could occur between internal cavity and an accessible region.[12] (Figure S4) Such inaccuracy mainly comes from the imbalance between data distribution of the exterior and interior. (Figure S1)

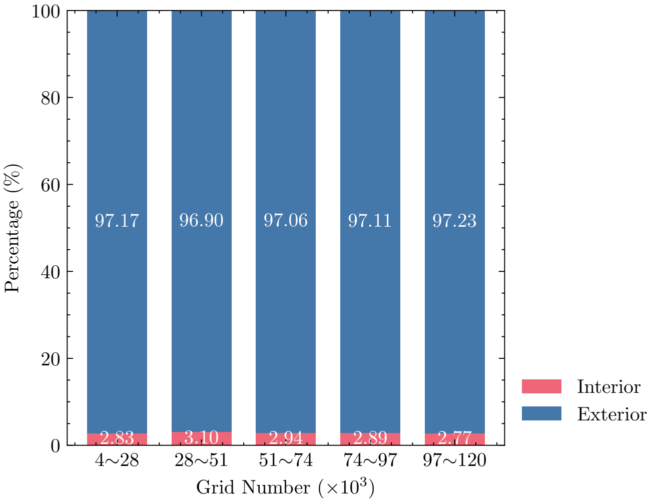

Figure 4: Percentage of interior points within the protein dataset.

Comments from review #4:

What is the PB solver used in pairing with GENIUSES?

Response:

Thank you for inquiring about the specific PB solver paired with our GENIUSES model. For our implementation, we utilize the Conjugate Gradient solver.

Comments from review #4:

In the robustness section, the Authors seem to be surprised that a model trained on proteins performs well also on nucleic acids and protein complexes, why? The construction of the SES is agnostic with respect to chemistry, it starts from a union of partially overlapping balls. So, since the radii of the atoms are similar and also the bond lengths are similar, also the surface should be predicted in a similar way, or not?

Response:

Definitely, we agree that if the radii of the atom, the bond length, and spatial distribution are similar, the surface generated or predicted should be similar. However, some announced differences should be noted between proteins and nucleic acids. For example, the structural flexibility and pattern are quite different. For protein complexes, the inter-atomic distances between interacting monomers are quite different from those within monomer structures.

Revision made in text: (Page 11)

The robustness of our model is initially demonstrated through its transferability. Our GENIUSES model, trained exclusively on a protein dataset, is subsequently applied directly to surface generation for both nucleic acid and protein complex datasets where the structural flexibility and pattern are quite different from training ones.

Comments from review #4:

This sentence seems inconsistent: "For systems containing fewer than 2000 atoms, regardless of the platform or library utilized, our method significantly outperforms the classical SES and MLSES. However, when the atom count exceeds 2000, only the method implemented with Kernel Fortran on a CPU platform exhibits a marginally slower speed than MLSES implemented on GPU."

Response:

Sorry for the confusion. What we want to stress is the superior performance of GENIUSES over both classical SES and MLSES when systems are smaller than 2000 atoms. When systems are larger than 2000 atoms, the conclusion still holds except for GENIUSES implemented on the CPU platform. To improve readability, we have redrawn Figure 4 and additional statements were added to the main text.

Revision made in text: (Page 14)

For systems containing fewer than 2000 atoms, regardless of the platform or library utilized, our method (solid circle) significantly outperforms the classical SES (blue "X") and MLSES (pink "X"). When the atom count exceeds 2000, most implementations of the method still outperforms the classical SES (blue "X") and MLSES (pink "X"), except that implemented with the Kernel Fortran on CPU platform (green solid circle), which exhibits a marginally slower speed than MLSES (pink "X").

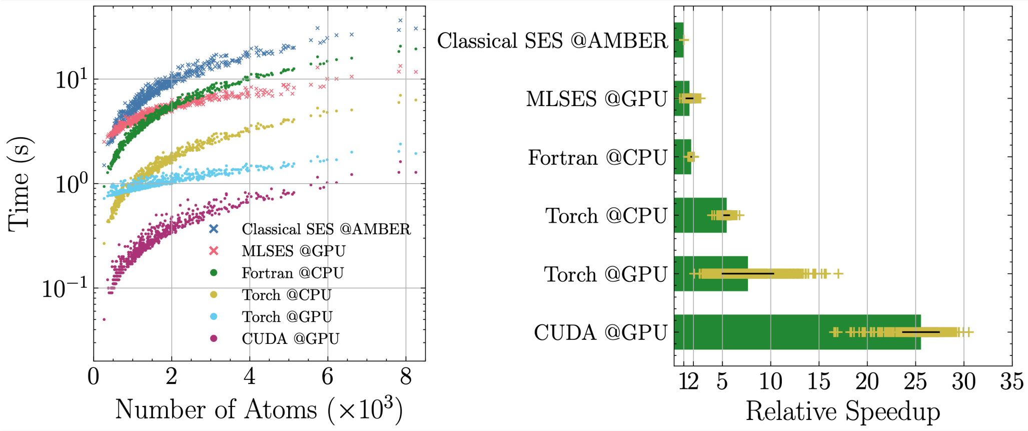

Figure 5: Comparative analysis of performance with average run time and relative speedup for the protein dataset. (a) Run time as a function of number of atoms using different methods and GENIUSES implemented on different platforms; GENIUSES related methods are all represented in solid circles, while Classical SES and MLSES are in the style of "X" (b) Relative speedup with respect to the Classical SES.

Comments from review #4:

I would not say "grid size of 0.35Å" but "grid spacing of 0.35Å"

Response:

Thank you for the clarification. We have amended the manuscript to reflect this change and ensure consistency in the usage throughout.

==========The end of reply to reviewer #4 ============

References

[1] S Osher and R Fedkiw. Level set methods and dynamic implicit surfaces springer. *New York Berlin Heidelberg*, 2003.

[2] Xiang Ye, Jun Wang, and Ray Luo. A revised density function for molecular surface calculation in continuum solvent models. *Journal of chemical theory and computation*, 6(4):1157–1169, 2010.

[3] RJ Zauhar and RS Morgan. Computing the electric potential of biomolecules: application of a new method of molecular surface triangulation. *Journal of Computational Chemistry*, 11(5):603–622, 1990.

[4] Frank Eisenhaber and Patrick Argos. Improved strategy in analytic surface calculation for molecular systems: Handling of singularities and computational efficiency. *Journal of Computational Chemistry*, 14(11):1272–1280, 1993.

[5] Tony You and Donald Bashford. An analytical algorithm for the rapid determination of the solvent accessibility of points in a three-dimensional lattice around a solute molecule. *Journal of Computational Chemistry*, 16(6):743–757, 1995.

[6] Walter Rocchia, Sundaram Sridharan, Anthony Nicholls, Emil Alexov, Alessandro Chiabrera, and Barry Honig. Rapid grid-based construction of the molecular surface and the use of induced surface charge to calculate reaction field energies: Applications to the molecular systems and geometric objects. *Journal of computational chemistry*, 23(1):128–137, 2002.

[7] Qin Cai, Xiang Ye, Jun Wang, and Ray Luo. On-the-fly numerical surface integration for finite-difference poisson–boltzmann methods. *Journal of chemical theory and computation*, 7(11):3608–3619, 2011.

[8] Lin Li, Chuan Li, Zhe Zhang, and Emil Alexov. On the dielectric "constant" of proteins: smooth dielectric function for macromolecular modeling and its implementation in delphi. *Journal of chemical theory and computation*, 9(4):2126–2136, 2013.

[9] Jun Wang, Qin Cai, Ye Xiang, and Ray Luo. Reducing grid dependence in finite-difference poisson–boltzmann calculations. *Journal of chemical theory and computation*, 8(8):2741–2751, 2012.

[10] Michael L Connolly. Solvent-accessible surfaces of proteins and nucleic acids. *Science*, 221(4612):709–713, 1983.

[11] Dong Xu and Yang Zhang. Generating triangulated macromolecular surfaces by euclidean distance transform. *PloS one*, 4(12):e8140, 2009.

[12] Sergio Decherchi and Walter Rocchia. A general and robust ray-casting-based algorithm for triangulating surfaces at the nanoscale. *PloS one*, 8(4):e59744, 2013.

[13] David A Case, Nikolai R Skrynnikov, Thomas E Cheatham III, Oleg Mikhailovskii, Carlos Simmerling, Yi Xue, Adrian Roitberg, Yi Xue, Adrian Roitberg, Sergei A Izmailov, Kenneth M Merz, Koushik Kasavajhala, et al. *AMBER 23 Reference Manual.* University of California, 2023.

[14] Michael L Connolly. Analytical molecular surface calculation. *Journal of applied crystallography*, 16(5):548–558, 1983.

[15] Byungkook Lee and Frederic M Richards. The interpretation of protein structures: estimation of static accessibility. *Journal of molecular biology*, 55(3):379–IN4, 1971.

[16] Lubor Ladicky, Olivier Saurer, SoHyeon Jeong, Fabio Maninchedda, and Marc Pollefeys. From point clouds to mesh using regression. In *Proceedings of the IEEE International Conference on Computer Vision*, pages 3893–3902, 2017.

[17] Charles R Qi, Hao Su, Kaichun Mo, and Leonidas J Guibas. Pointnet: Deep learning on point sets for 3d classification and segmentation. In *Proceedings of the IEEE conference on computer vision and pattern recognition*, pages 652–660, 2017.

[18] Panos Achlioptas, Olga Diamanti, Ioannis Mitliagkas, and Leonidas Guibas. Learning representations and generative models for 3d point clouds. In *International conference on machine learning*, pages 40–49. PMLR, 2018.
